# Molecular modelling and *de novo* fragment-based design of potential inhibitors of beta-tubulin gene of *Necator americanus* from natural products

**DOI:** 10.1016/j.imu.2021.100734

**Published:** 2021-09-15

**Authors:** Odame Agyapong, Seth O. Asiedu, Samuel K. Kwofie, Whelton A. Miller, Christian S. Parry, Robert A. Sowah, Michael D. Wilson

**Affiliations:** a Department of Biomedical Engineering, School of Engineering Sciences, College of Basic & Applied Sciences, University of Ghana, PMB LG 77, Legon, Accra, Ghana; b Department of Parasitology, Noguchi Memorial Institute for Medical Research (NMIMR), College of Health Sciences (CHS), University of Ghana, P.O. Box LG 581, Legon, Accra, Ghana; c West African Centre for Cell Biology of Infectious Pathogens, Department of Biochemistry, Cell and Molecular Biology, College of Basic and Applied Sciences, University of Ghana, Accra, Ghana; d Department of Medicine, Loyola University Medical Center, Maywood, IL, 60153, USA; e University of Pennsylvania, School of Engineering and Applied Science, Department of Chemical and Biomolecular Engineering, Philadelphia, PA, 19104, USA; f Center for Sickle Cell Disease, And Department of Microbiology, Howard University, Washington, DC, 20059, USA; g Department of Computer Engineering, School of Engineering Sciences, College of Basic & Applied Sciences, University of Ghana, Legon, Accra, Ghana

**Keywords:** *Necator americanus*, Anthelmintics, Natural products, Pharmacoinformatics, Molecular modelling, Drug discovery

## Abstract

The emergence of drug resistance against the known hookworm drugs namely albendazole and mebendazole and their reduced efficacies necessitate the need for new drugs. Chemically diverse natural products present plausible templates to augment hookworm drug discovery. The present work utilized pharmacoinformatics techniques to predict African natural compounds ZINC95486082, ZINC95486052 and euphohelionon as potential inhibitory molecules of the hookworm *Necator americanus* β tubulin gene. A library of 3390 compounds was screened against a homology-modelled structure of β tubulin. The docking results obtained from AutoDock Vina was validated with an acceptable area under the curve (AUC) of 0.714 computed from the receiver operating characteristic (ROC) curve. The three selected compounds had favourable binding affinities and were predicted to form no interactions with the resistance-associated mutations Phe167, Glu198 and Phe200. The compounds were predicted as anthelmintics using a Bayesian-based technique and were pharmacologically profiled to be druglike. Further molecular dynamics simulations and MM-PBSA calculations showed the compounds as promising anthelmintic drug leads. Novel critical residues comprising Leu246, Asn247 and Asn256 were also predicted for binding. Euphohelionon was selected as a template for the *de novo* fragment-based design of five compounds labelled A1, A2, A3, A4 and A5; with four of them having SAscore values below 6, denoting easy synthesis. All the five *de novo* molecules docked firmly in the binding pocket of the β tubulin with no binding interactions with the three known resistance mutation residues. Binding energies of −8.2, −7.6, −7.3, −7.2 and −6.8 kcal/mol were obtained for A1, A2, A3, A4 and A5, respectively. The identified compounds can serve as treasure troves from which future potent anthelmintics can be designed. The current study strives to assuage the hookworm disease burden, especially making available molecules with the potential to circumvent the chemoresistance.

## 1. Introduction

Hookworm infection remains a significant health burden globally. The disease is caused by the hookworm, an intestinal parasitic worm, that infects over 600 million persons especially in resource-limited countries and results in 135,000 deaths annually [1,2]. The hookworm infection belongs to a larger group of diseases called Neglected Tropical Diseases (NTDs) known to cause debilitating effects. Some NTDs are caused by parasitic worms including schistosomes, filarial and guinea worms [3]. Areas largely affected by hookworm include countries in sub-Saharan Africa, South Asia, Latin America, the Caribbean, the Middle East, and North Africa [4]. The human hookworm infection is primarily caused by *Ancylostoma duodenale* and *Necator americanus* [5] with the latter accounting for more than 85% of all hookworm infections [5,6]. The infection is known to affect growth as well as limit memory and cognition in children [7].

The β (beta) tubulin gene of the hookworm is critical for intracellular processes, cellular division, overall mobility, entry and migration of the parasite in the host cell. It is a subunit of the microtubule which, plays a crucial role in cell division and maintenance of the cytoskeleton [8]. It binds to two molecules of guanosine-5- triphosphate (GTP), at the positive end of microtubules [9]. Beta tubulin has so far been extensively exploited as a crucial target for anthelmintic and as a target for several other compounds [8]. Treatment using benzimidazoles including albendazole and mebendazole selectively target the beta-tubulin gene isotype, disrupting the polymerization of microtubules. Benzimidazoles confer their anthelminthic effect on susceptible nematodes by binding to their beta-tubulin gene resulting in the prevention of microtubule polymerization causing the destabilization of the intracellular processes and cellular division within the parasite and an overall immobility effect [10]. Community-based treatment methods normally employ a mass drug administration (MDA) strategy involving a combination regimen of albendazole, Ivermectin (IVM), and praziquantel (PZQ) [11–14]. This control strategy is, however, considered a high-risk approach in both humans and animals since it rather induces resistance [11]. For instance, there are reported cases of drug resistance in livestock in parts of Mali, Australia, South Africa and Paraguay [11]. Drawing lessons from this, it is therefore recommended a careful use of human anthelminthic in a way that will allow the prolonged use of these drugs and at the same time manage the problem of drug resistance and treatment failures.

Genetic mutations in the beta-tubulin gene are among the factors that have been reported to confer resistance in several parasitic nematodes including local strains of *N*. *americanus* [15,16]. These mutations occur at codons 167, 198 and 200 result in amino acid changes [15,17,18]. The changes lead to the substitution of phenylalanine with tyrosine at codons 167 and 200, and glutamate with alanine at amino acid position 198 [17,19,20]. Therefore, the binding and stabilization by alternative small molecule inhibitors with these residues Glu198, Phe167 and Phe200 at the active site of the beta-tubulin molecule is highly undesirable since it can lead to a decrease in binding affinity [21]. There is the need for molecules with different modes of binding.

Natural products (NPs) are structurally and chemically diverse compared to synthetic libraries [22]. Studies have shown that more than 50% of NPs pass the Lipinski rule of five for drug-likeness [23]. This makes natural products more absorbable than their synthetic counterparts do. Recent studies have reported a group of Dichapetalin compounds notably Dichapetalin A with promising anthelminthic activity against *N. americanus* [24].

*In silico* strategies for drug discovery presents an advantage in terms of time and cost for identifying novel chemotherapeutic agents by exploring a compendium of small compounds retrieved from freely available public databases. These methods have been used to identify some promising therapeutics against diseases [25,26]. Some of these databases include the African natural product database (AfroDB) and the North African Natural Product database (NANPDB) which contains a diverse and highly potent chemical landscape of natural compounds that could be explored for potential anthelminthic leads [27,28].

We sought to identify potential novel anthelminthic leads by performing virtual screening of natural products of African origin against a modelled 3D structure of beta-tubulin receptor of *N. americanus.* Also, we aimed at generating novel molecules using *de novo* fragment-based drug design. We characterized the mechanisms of binding for the resistance-associated mutations within the active site. In addition, pharmacological profiling, and relevant biological activity predictions were performed to determine the therapeutic utility of the potential lead molecules.

## 2. Methodology

A series of computational applications and techniques were employed in this study (Supplementary Table 1).

### 2.1. Template selection and homology modelling of N. americanus beta-tubulin gene

A search in Protein Data Bank (PDB; http://www.rcsb.org/) revealed no solved tertiary structure of *N. americanus* beta tubulins, hence the primary sequence of the protein (Gene ID: NECAME_11014, Accession number: W2T758) was retrieved from UniProt [29]. For template identification, Aguayo-Ortiz et al. employed the Iterative Threading ASSEmbly Refinement (I-TASSER) software tool to predict a suitable template based on a binding site containing residues associated with mutations [30]. Using a similar approach, our protein sequence comprising of 449 amino acids was submitted for template and binding site identification via the Iterative Threading ASSEmbly Refinement (I-TASSER) server [31], which predicted four plausible templates. The D chain of the subunit of the multimeric structure of tubulin tyrosine ligase (T2R-TTL) [PDB ID: 5C8Y], was selected for further studies due to the presence of amino acid residues associated with resistance as well as a high sequence identity to the template. The crystallographic structure of T2R-TTL (PDB ID: 5C8Y, resolution: 2.59 Å) was downloaded from PDB and used as a template in homology modelling of the beta-tubulin receptor using MODELLER v 9.17 [32]. The *align2d* function in MODELLER v9.17 was used to align the sequence of the target with the template and five candidate 3-D models generated using MODELLER v 9.17. The best model was then chosen based on the lowest value of the Discrete Optimized Potential Energy (DOPE) and high GA341 scores [32]. The selected best model and the template were structurally aligned using SuperPose [33].

### 2.2. Model refinement and assessment

The selected best model was refined to fix steric clashes and bumps using the WHAT IF server [34]. This is due to models having undesired bond lengths, bond angles, torsion angles and contacts. The refined structure was then energy minimized using GROMOS43B1 force field in Swiss-PdbViewer v 4.10 [35] to correct local bond and angle geometry and to relax the close contacts in the geometric chain. The WHAT IF program implements WHAT_CHECK to ascertain and fix steric clashes based on the overlap of two non-bonding atoms of distance cutoff set at 0.4 Å. The minimized model was visualized with PyMOL v 1.74 [36] and further optimized using molecular dynamics simulations. This was performed to evaluate the overall stability, folding, and obtain insight into the conformational changes as well as the dynamics of the refined model. The molecular dynamics (MD) simulation of the structure was performed with the Linux version of GROMACS v 5.1.4 [37] software package by utilizing the GROMOS 96_43a1 force field and the simple point charge (SPC) water model by passing the “-water spce” command. The modelled structure was first immersed in a periodic water box of cubic shape (1 nm thick). After solvating the receptor, the net charge on the protein was +8e. Genion command in GROMACS was used to add eight chloride (Cl-) counter ions to neutralize the net charge on the protein. Electrostatic energy was computed via the particle mesh Ewald method with a computational load of 0.19. The cutoff distance for the calculation of the Coulomb and van der Waals interaction was 1.0 Å with the cutoff scheme being Verlet for minimization of 50000 steps. The system was subjected to a two-step ensemble process (NVT and NPT) at a temperature of 300 K and pressure of 1 bar (P) for 2 ps. Linear Constraint Solver (LINCS) constraints were performed for all bonds, with position restraint on the protein and allowing only the water molecules to move to equilibrate with the protein structure. The final production run was performed for the minimization of 5 ns under the same equilibration conditions of 300 K and 1 bar. The results were analyzed using GROMACS v 5.1.4 and GRACE v 5.1.25 [38] which uses the command xmgrace in a Linux terminal. The last frame of the minimized protein in gro format was saved as a pdb format using Visual Molecular Dynamics (VMD) v1.9.3 [39]. The minimized model was then validated by generating a Ramachandran plot [40] using the PROCHECK v 3.5.4 [41]. Other programs such as ERRAT, VERIFY3D and Qmean [42,43] were also used to corroborate the PROCHECK results.

### 2.3. Prediction and analysis of binding site

The potential binding site of the beta-tubulin model containing the amino acid residues of interest was predicted using the Computed Atlas of Surface Topography of proteins (CASTp) [44] and further visualized in PyMOL [36].

### 2.4. Protein preparation for docking

AutoDockTools v 4.2.6 [45] was used in the preparation of the protein model which involved the assignment of Gasteiger partial charges [46]. All existing water or solvent molecules were removed to eliminate the influence of solvent interactions in the protein-ligand docking. The receptor file saved in Protein Data Bank partial charge and atom type (pdbqt) format was used as an input receptor file for AutoDock Vina.

### 2.5. Selection and preparation of ligands

**Fig. 1.**
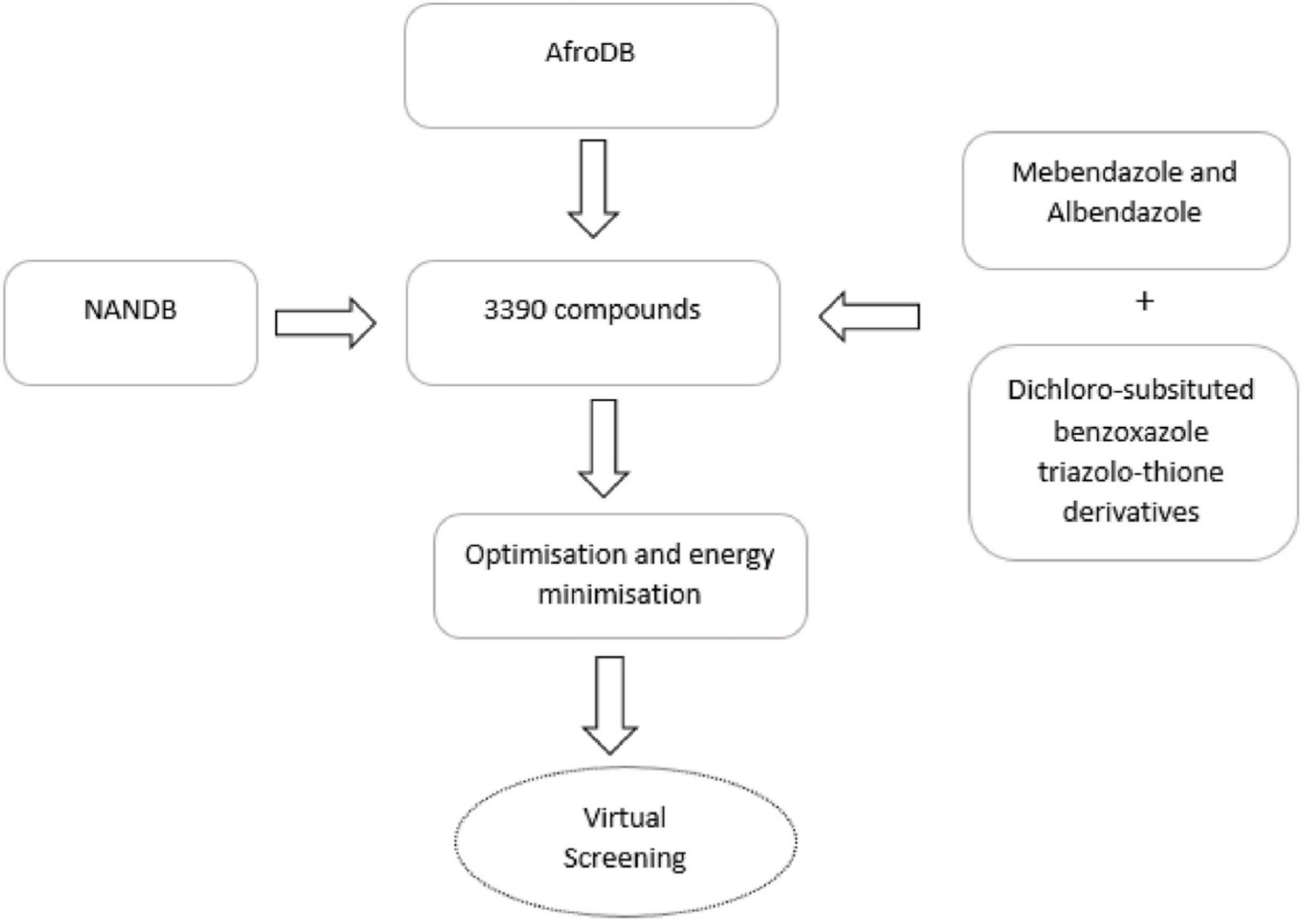
A flow chart for the preparation and selection of ligands for virtual screening. The library consisted of African natural products and five known hookworm beta-tubulin inhibitors.

An integrated natural product library (Fig. 1) comprising 885 compounds from the African Natural Product Database (AfroDb) [28], a subset of ZINC [47] and 2500 compounds from the North African Natural Product Database (NANPDB) [27] databases were retrieved in Structural Data Format (sdf). The two libraries contain structurally diverse natural products of African origin [27,28]. Additionally included in the screening dataset were two known anthelmintics namely albendazole and mebendazole [48–50] as well as three potent dichloro-substituted benzoxazole-triazolo-thione derivatives namely 6, 8-dichloro [1,2,4]triazolo[3,4-b] [1,3]benzoxazole-3(2h)-thione (PubChem ID 53327690); 5,7-dichloro-1,3-Benzoxazole-2-thiol (PubChem IDs 723308); and 6,8-dichloro-2-{[(4nitrophenyl)amino]methyl} [1,2, 4]triazolo[3,4b] [1,3]benzoxazole-3(2h)-thione (PubChem ID 53327692) [51]. The structures of the controls are provided in the Supplementary Figure 1. The sdf files of the five compounds were retrieved via PubChem, a freely available chemical structure database [52]. The total set of 3390 compounds were further optimized and the energy was minimized using Open Babel 2.3.1 within the Pyrx 0.8 interface [53] with MMFF94 force field energy minimization. All ligand files were then converted to compatible pdbqt files using AutoDock Tools.

### 2.6. Virtual screening

AutoDock Vina [54] tool in PyRx v 0.8 [55] was used to virtually screen a library of 3390 compounds against the binding pocket of the predicted model. The grid box with dimensions (22.5 Å × 22.5 Å x 22.5 Å) and centre (−18.35 Å, −8.23 Å, −22.48 Å) was set around the predicted active site residues of the receptor. A default exhaustiveness of 8 was used. After completion of the virtual screening process, the top hits, with the lowest binding energies (kcal/mol) relative to the known inhibitors, were selected for further visualization in PyMOL [36]. The top 30 hits with reasonably good binding energies and binding poses were selected for further evaluation.

### 2.7. Docking protocol validation

Five molecules comprising albendazole and mebendazole, as well as three potent dichloro-substituted benzoxazole-triazolo-thione derivatives with PubChem CIDs [52] 53327690, 723308 and 53327692 were used as actives in the validation of docking protocol. Albendazole and mebendazole are broad-spectrum anthelmintic drugs targeting beta-tubulin [48,50]. Moreover, the choice of including the three dichloro-substituted benzoxazole-triazolo-thione derivatives was based on their reported efficacious inhibitory activities against helminth beta tubulins [51]. The SMILES of the five molecules were used to obtain a dataset of decoys via the Directory of Useful Decoys- Enhanced (DUD-E) [56]. Fifty decoys were obtained for each ligand, then each set of decoys has similar physicochemical properties to the parent ligand but is dissimilar in topology [57]. Furthermore, the dataset of 255 compounds comprising of 250 decoys and 5 ligands were screened against the predicted active site of the modelled beta tubulin receptor using AutoDock Vina. The docking result containing the respective binding energies of the ligands and their decoys was used to compute the Area Under the Curve (AUC) of the Receiver Operating Curve (ROC) via easyROC v 1.3.1 [58]. The variables were set to “nonparametric” as the method for curve fitting, “DeLong (1988) [+]”as the method for standard error estimation and confidence interval, and a “Type I” error of 0.05.

### 2.8. Protein-ligand interaction profiling

Ligplot+ [59] was used to study the 2D protein-ligand interaction which includes the hydrogen bonding and the hydrophobic contacts. The best poses of the hits were saved in a ‘.pdb’ file format. The protein-ligand complex for these poses was visualized using PyMOL. The complexes were then loaded into the Ligplot+ workspace to generate the 2D schematic intermolecular interactions between the protein and the ligands.

### 2.9. Exploring the anthelmintic activity of the hits

Prediction of Activity Spectra for Substances (PASS) [60] which a is Bayesian-based machine learning technique was used to predict the anthelmintic activity of the top 30 hits. Anthelmintics are drugs for the treatment of helminth infections, including hookworm infection. A DrugBank and literature search for anthelmintic activity was also done to support the PASS predictions based on the structural information of the hits.

### 2.10. Drug likeness and pharmacological profiling

The SMILES of the hits were used to obtain the pharmacological profiles and physicochemical properties of the PASS predicted anthelmintic hits via SwissADME [61]. Parameters considered include molecular weight, number of hydrogen bond acceptors and donors for evaluating drug-likeness and gastrointestinal absorption, P-gp substrates, and cytochrome P450 inhibition for evaluating the pharmacokinetics.

### 2.11. Toxicity prediction analysis

The toxicity profiles of the PASS anthelmintic predicted compounds were evaluated and analyzed using the OSIRIS property explorer in DataWarrior v 4.5.2 [62]. OSIRIS explorer uses a precomputed set of known toxic compounds and fragments to predict relevant properties comprising tumorigenicity, mutagenicity reproductive effect and irritancy [62]. ADVERPred [63] was also used to predict the nephrotoxicity and hepatotoxicity of the compounds.

### 2.12. Molecular dynamics of protein-ligand complexes and MM-PBSA analyses

The MD simulations of the protein-ligand complex were performed in GROMACS v 2018 [64]. The protein topology of the modelled beta-tubulin was generated using the GROMOS 96_43a1 force field whereas the ligand topologies were generated via PRODRG [65], with defined settings (Chirality: Yes, Charges: Full, and EM: No). A protein-ligand complex was then constructed. The complex was solvated with SPC water in a cubic box of size 1.0 nm and neutralized with Na and Cl ions. Energy minimization of the complex was conducted for 50,000 steps using the steepest descent algorithm. The ligands were restrained before the NVT and NPT ensemble simulations. Equilibration simulation was run for 100 ps for each ensemble. A production MD of 100 ns simulation was performed on the complexes. The g_mmpbsa [66] was used to compute the free binding energies of the complexes over the 100 ns simulation using the **molecular mechanics Poisson-Boltzmann surface area (MM-PBSA) method**. R programming package was used to generate graphs for the MM-PBSA simulation.

### 2.13. De novo design of inhibitors

A potential lead complex was used as input for the *de novo* design of novel ligands via e-LEA3D [67]. A binding site radius of 10 Å and a weight in a final score of 1 were selected in setting up the docking function. The coordinates of the active site as used in molecular docking studies was considered. The maximum number of conformers, number of generations and population size were set at 1, 30 and 30, respectively.

## 3. Results and discussion

### 3.1. Template identification, homology modelling of proteins, molecular dynamic simulations and validation

**Fig. 2.**
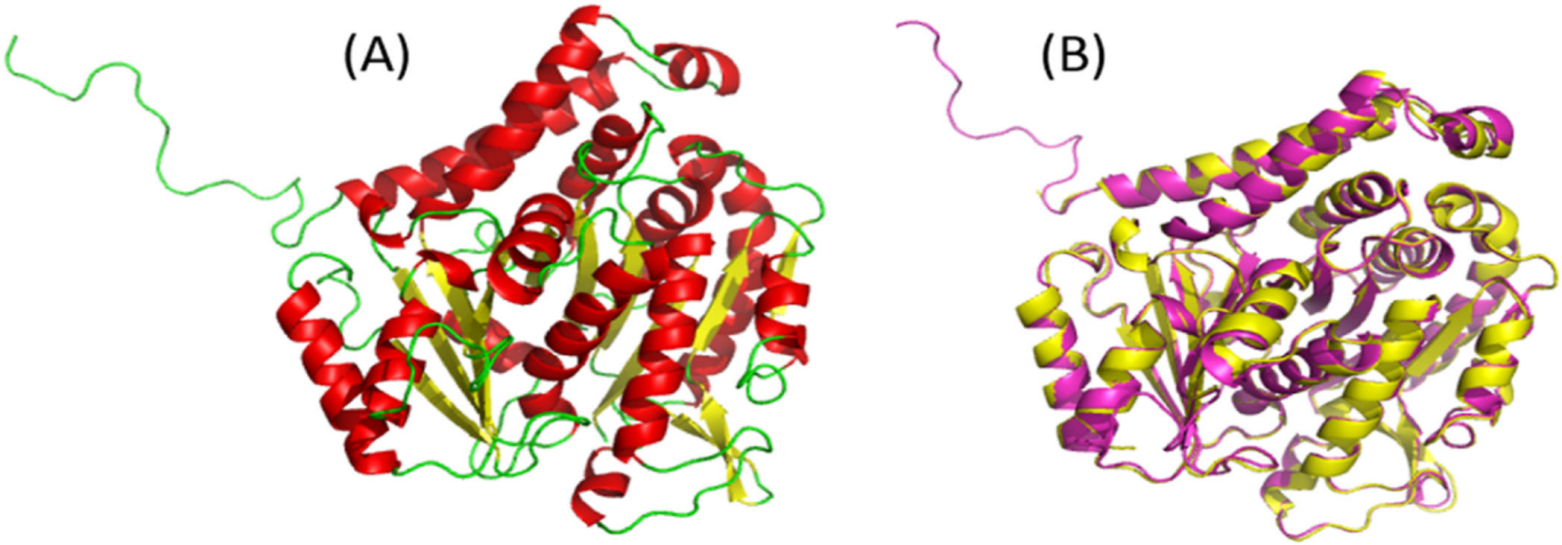
(A) 3D model of the beta-tubulin of *N. americanus* (UniProt ID, W2T75). The helices are shown in red, beta sheets in yellow and loops in green. (B) Structural alignment between the protein model and template generated using SuperPose and visualized in PyMOL. The model is shown in purple and the template in yellow. The superposition of the two structures had an acceptable RMSD of 1.32 Å in SuperPose. (For interpretation of the references to colour in this figure legend, the reader is referred to the Web version of this article.)

Aguayo-Ortiz et al. used the crystal structure of *Ovis aries* beta-tubulin as a template to generate the homology model of the beta-tubulin of the nematode *Trichinella spiralis* and to predict a binding site that contained conserved residues [30]. Similarly, using I-TASSER [31,68], the D chain of the crystal structure of tubulin tyrosine ligase (T2R-TTL) with PDB ID 5C8Y was chosen as the best template for homology modelling based on the presence of conserved amino acid residues Phe167, Glu198 and Phe200, within the binding site. A sequence alignment with the template also showed that the beta-tubulin is homologous to the subunit of the T2R-TTL with 88% sequence identity and 94.3% sequence similarity (Supplementary Figure 2). In addition, the results of sequence alignment revealed that the predicted active site contained residues Phe167 and Glu198. Model 5 was selected as the best structural model of the beta-tubulin of *N. americanus* (UniProt ID W2T75) based on the lowest discrete optimized potential energy (DOPE) score of −53055.30859 obtained (Table 1). The DOPE score and GA341 are methods used for evaluating the accuracy and reliability of modelled protein structures. DOPE is an atomic distance-dependent statistical method that is useful in evaluating the energies of generated models [69]. Models with the lowest DOPE score and highest GA341 score are considered reasonably better. The resulting protein model is a monomer, folded into a β domain that consists of 11-stranded β-sheets, and 11 α-helices (Fig. 2A and B). Structural alignment of the 3D model and template using SuperPose [33] gave a root mean square deviation (RMSD) of 1.32 Å. The RMSD value obtained indicates an acceptable difference between the protein model and the template [33]. In addition, the Modeller objective function (molpdf) was used to measure the sum of all the restrains.

The overall structure of the model is similar to the template protein as expected (Fig. 2B). Additionally, the structural alignment revealed conserved key amino acid residues at positions 167 and 198 but only a residue difference at position 200 where phenylalanine (aromatic non- polar residue) is present in the model structure but a tyrosine (aromatic polar residue) in the template; these two residues differ by only a hydroxyl group. Further, there were binding site residue differences at positions 165 and 166, where two serine residues (polar amino acid) were present in the model while asparagine and threonine (also polar amino acids) occupied those respective positions in the template. In addition, there were amino acid differences at positions 317 and 370, where methionine and valine (hydrophobic amino acid residues) were present in the model and isoleucine (also hydrophobic amino acid) at the respective positions in the template.

### 3.2. Model refinement and assessment

**Fig. 3.**
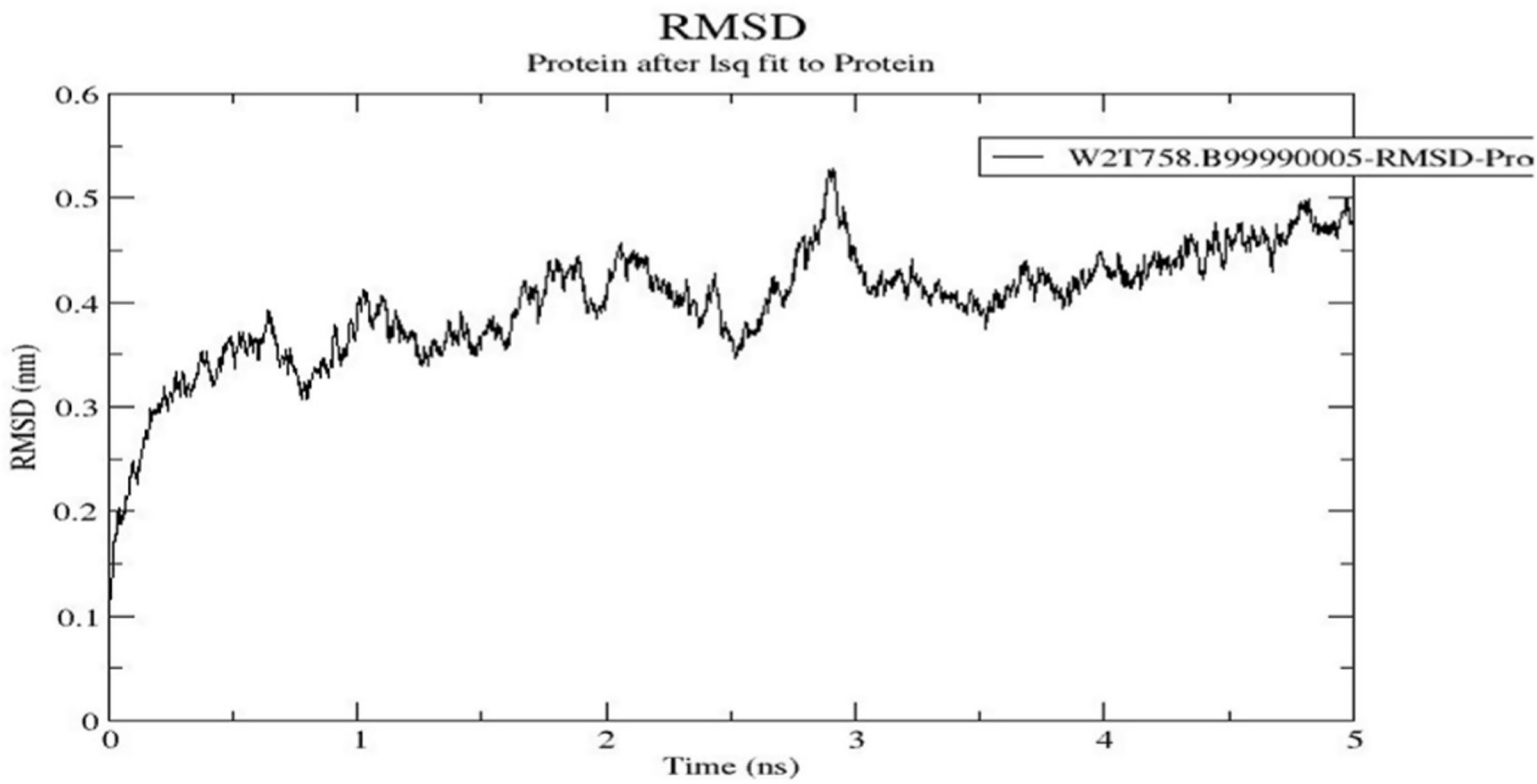
RMSD plot of the molecular dynamics simulation using GROMACS. A plot of RMSD in nanometres (nm) against time in nanoseconds (ns). The RMSD increased from 0 ns to 0.5 ns and levelled off with slight fluctuations towards the end of 1 ns.

Protein model refinement and energy minimization were performed on our best model using Swiss-PdbViewer [70] and GROMACS [64,71], respectively. Analysis of the GROMACS generated trajectories of the refined protein model indicated that the RMSD increased from the beginning but after a period of 0.5 ns, it remained relatively stable for the rest of the duration of the simulation (Fig. 3). This suggests that the model had very low RMSD for the backbone with less Root Mean Square (RMS) fluctuations and flexibility, and thus indicating that the model was well-conditioned.

**Fig. 4.**
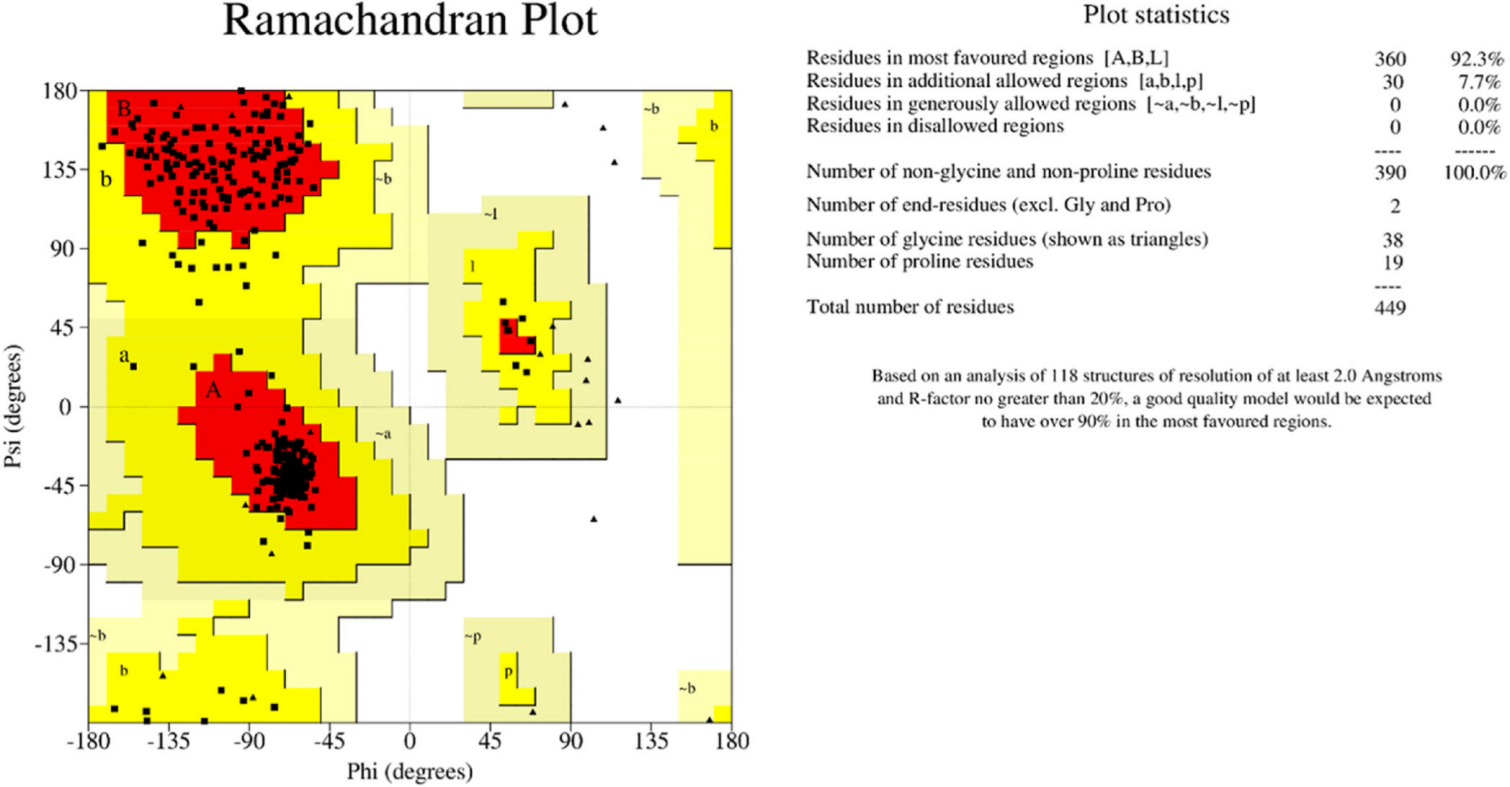
Ramachandran plot of beta-tubulin model from *N. americanus* obtained by PROCHECK: 92.3% residues in favourable regions (A, B, L); 7.7% residues in the additional allowed region (a, b, l, p); 0.0% residues in generously allowed regions (-a,-b,-p,-l); and 0% residues in disallowed regions.

The quality of the optimized structure was finally evaluated by generating a Ramachandran plot using PROCHECK [41]. Ramachandran plots highlight the most favoured, allowed, generously allowed and disallowed regions of the modelled protein structure. Ideally, a model of reasonably high quality should have at least 90% residues in the core regions [40]. The Ramachandran plot for the predicted model revealed that 92.3% of residues were within the most favourable region, whilst 7.7% were in the allowed region (Fig. 4), supportive that the predicted model was of reasonably high quality. In addition, the overall quality factor predicted by the ERRAT for the model was 89.327 (Supplementary Figure 3) which corroborates the quality of the protein model structure. ERRAT [72] provides the overall quality factor for non-bonded atomic interactions and the generally accepted value should be greater than 50 for a high-quality model. When the model was further validated using VERIFY 3D [42], 88.77% of the residues were predicted as having an average 3D-1D score greater than 0.2.

### 3.3. Prediction and analysis of binding site

**Fig. 5.**
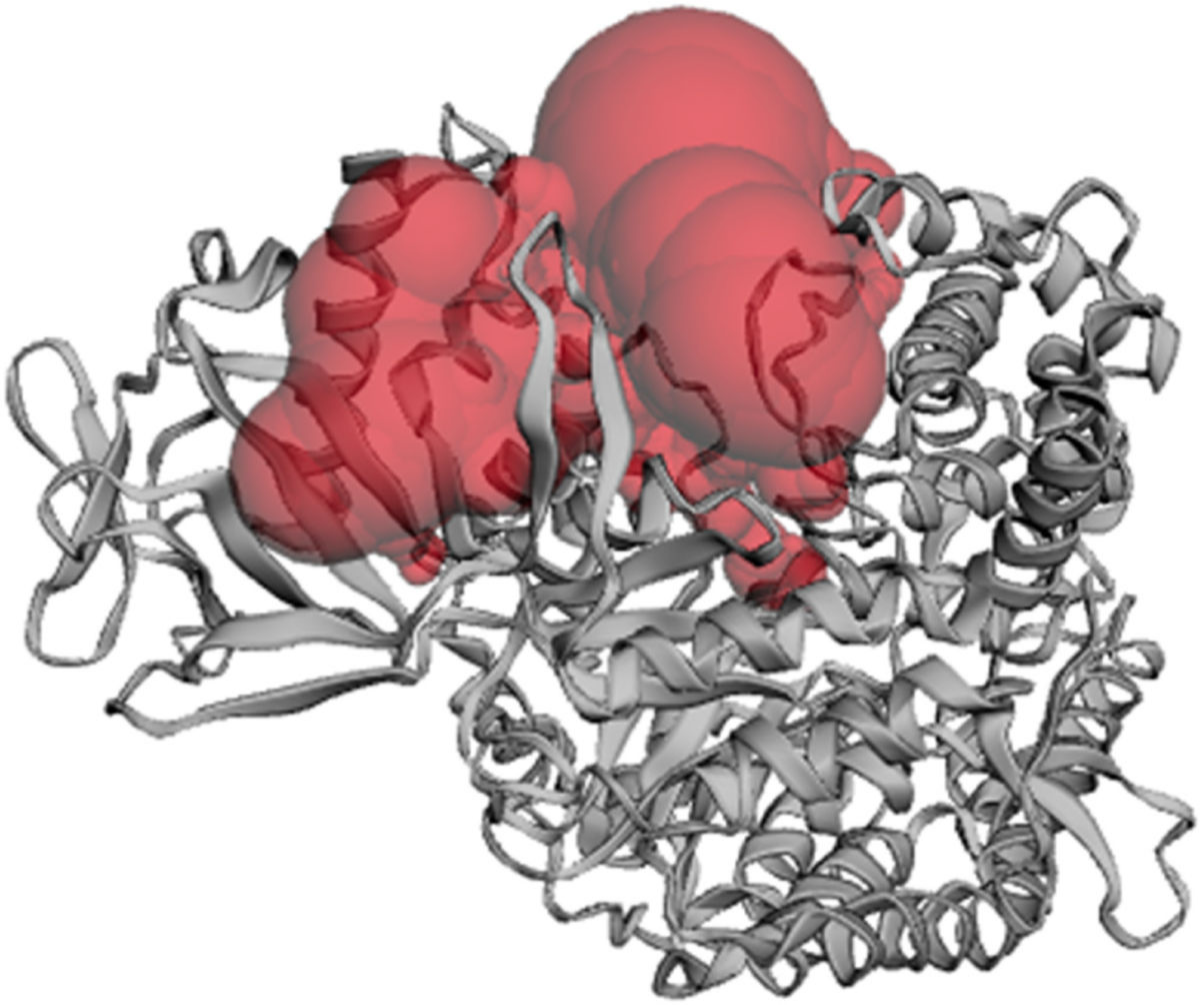
Predicted binding cavity of the modelled structure via CASTp. The predicted binding pocket is depicted as red. (For interpretation of the references to colour in this figure legend, the reader is referred to the Web version of this article.)

The residues within the binding site of tubulins generally have a high sequence and structural conservation among helminth tubulins which are different from other families of tubulins. The predicted binding pocket included all residues whose mutation are linked to anthelminthic resistance (Supplementary Figure 4) and had a computed area of 401.920 Å^2^ and volume of 164.250 Å^3^. Forty-three (43) residues formed the putative binding pocket and these included conserved residues Phe167, Glu198 and Phe200 (Fig. 5, Supplementary Figure 4).

### 3.4. Virtual screening

Molecular docking is a useful computational technique in informing the selection of lead compounds [73]. After screening an integrated library of 3390 against the active site, the top hits were selected for further analysis based on their binding energies. The docking poses of the top hits were further visualized using PyMOL. Hits that were not docked firmly in the active site pocket were ferreted out. The binding energies and docking poses were used as criteria to select 30 hits for further evaluation. These compounds had lower binding energies than the five known inhibitors. Table 2 shows the docking results of the top 30 hits ranked according to their binding energies. S,5Z,8Z,11Z,13E, 17Z)-15-hydroxy-1-(2,4,6- trihydroxyphenyl)-15- methylicosa-5,8,11, 13,17- pentaen-1-one had the lowest binding energy of −8.7 kcal/mol, suggesting a plausible strong molecular interaction. The binding energies of the top 30 hits ranged from −8.7 kcal/mol to −7.7 kcal/mol. Additionally, the screening results of the known hookworm beta-tubulin inhibitors namely albendazole and mebendazole and three other dichloro substituted benzoxazole-triazolo-thione derivatives were included (Table 2). Mebendazole had the lowest binding energy of −7.0 kcal/mol amongst the known inhibitors followed by the compound of PubChem ID 53327692 with a binding energy of −6.4 kcal/mol (Table 2). The docking results showed the top 30 hits having relatively lower binding energies than the known inhibitors.

### 3.5. Docking protocol validation

**Fig. 6.**
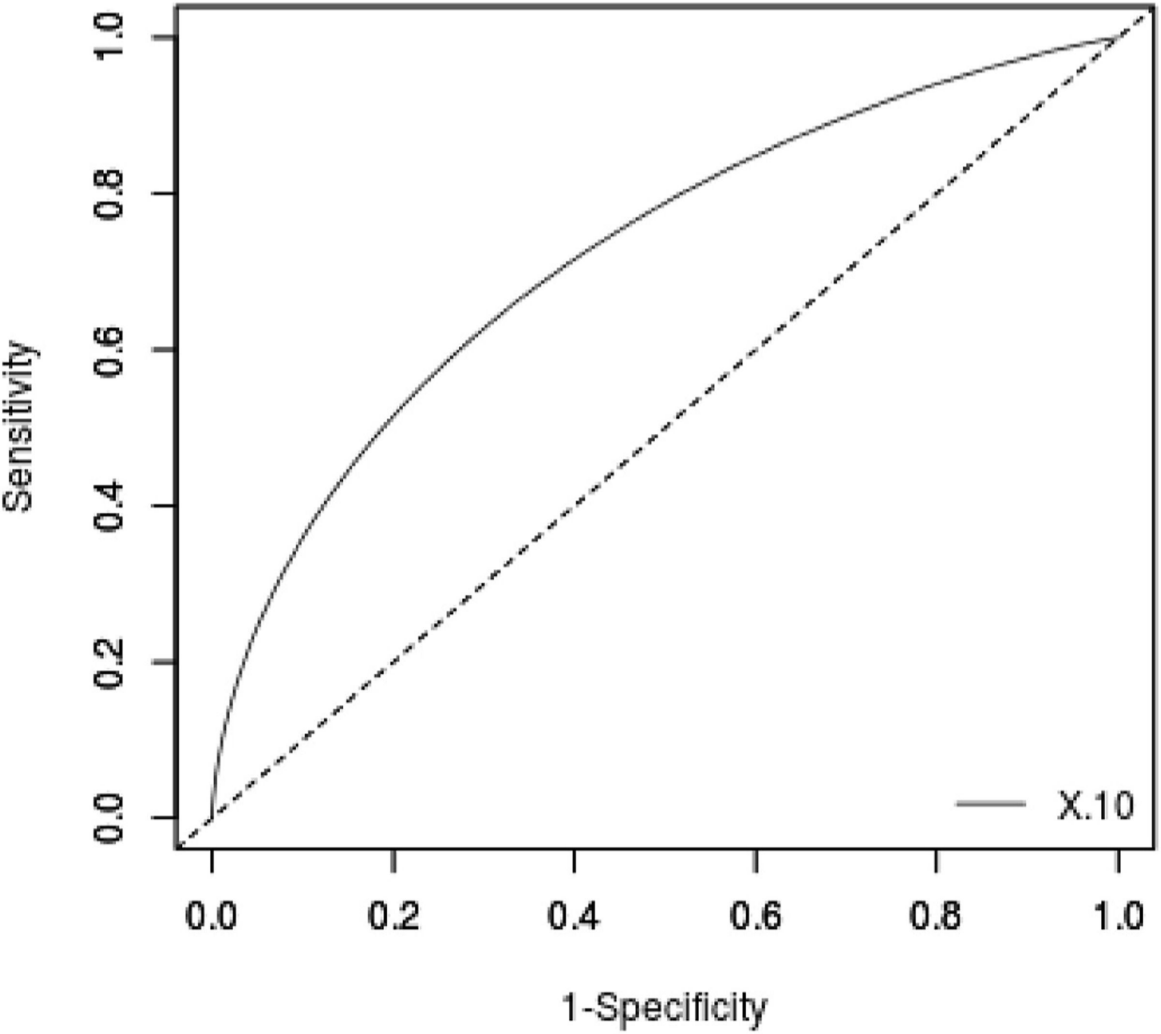
A ROC curve generated after screening 255 compounds consisting of five inhibitors and 250 decoys against the 3D model of the beta-tubulin of *N. americanus.* An acceptasble AUC of 0.714 was obtained.

The docking protocol of AutoDock Vina [54] was validated using the ROC curve analysis. Five hookworm’s beta-tubulin inhibitory ligands comprising albendazole, mebendazole, and PubChem IDs 53327690, 723308 and 53327692 with their respective decoys were screened against the beta-tubulin model to generate the ROC curve. The ROC curve evaluates a docking model’s ability to discriminate between actives and decoys [74]. An area under the curve (AUC) value of 1 is considered a perfect classification, and below 0.5 is poor discrimination [75,76]. Accordingly, an AUC value of 0.7–0.8 is considered acceptable and an AUC value of 0.8–0.9 is interpreted as excellent. Moreover, an AUC value of above 0.9 is considered outstanding [75,77]. By screening 5 actives and 250 decoys against hookworm beta-tubulin via AutoDock Vina, an AUC value of 0.714 was computed from the ROC curve (Fig. 6). The computed AUC value is considered acceptable [75].

To support the ROC evaluation of the AutoDock Vina, previously reported studies had employed AutoDock Vina to successfully screen compounds against helminth beta-tubulin receptors [51,78] The predicted compounds were experimented and confirmed as potent helminth inhibitors, suggesting that AutoDock Vina is an effective tool for deriving potential inhibitors for beta tubulins of *N. americanus*.

### 3.6. Characterization of the mechanism of binding

**Fig. 7.**
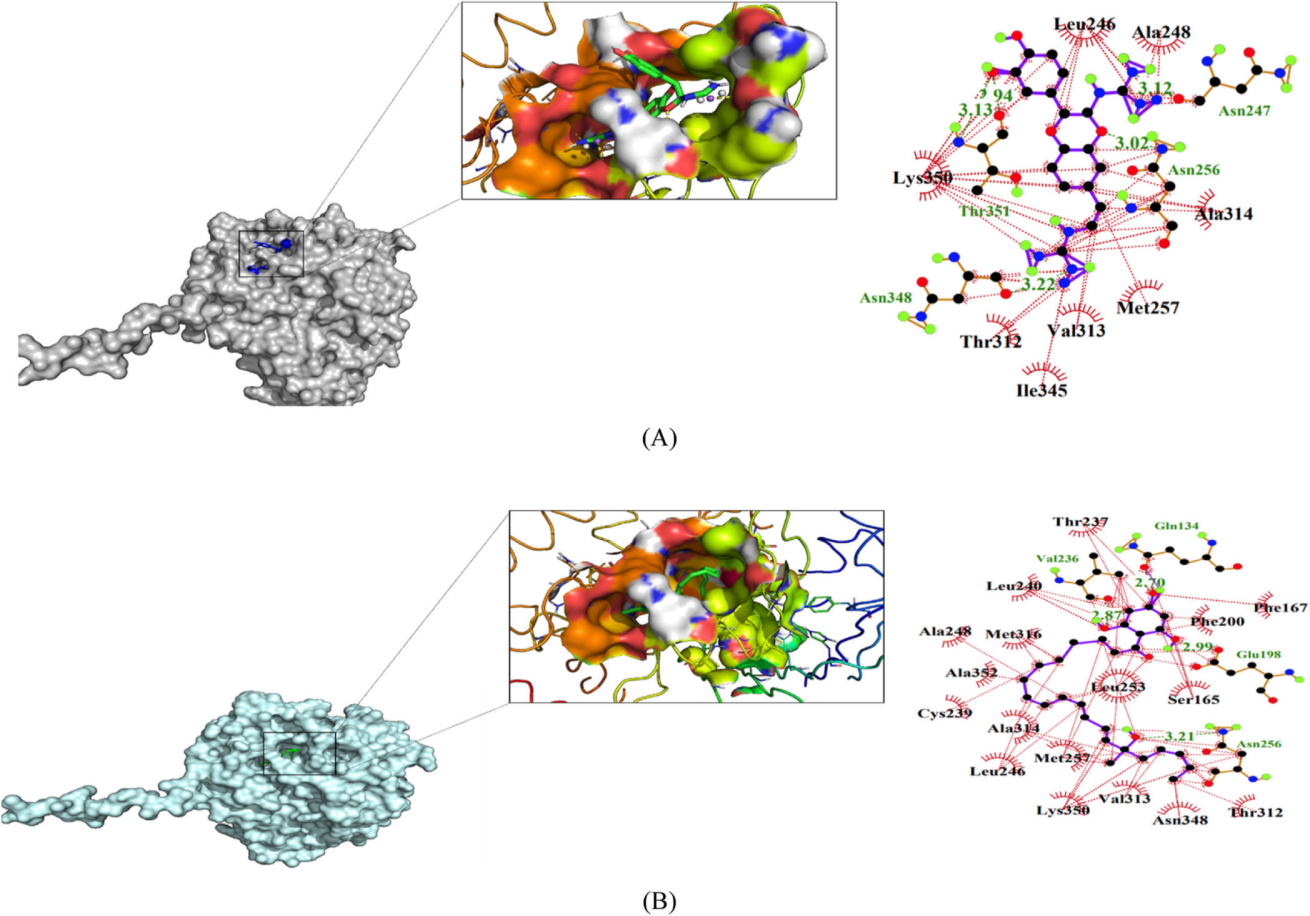

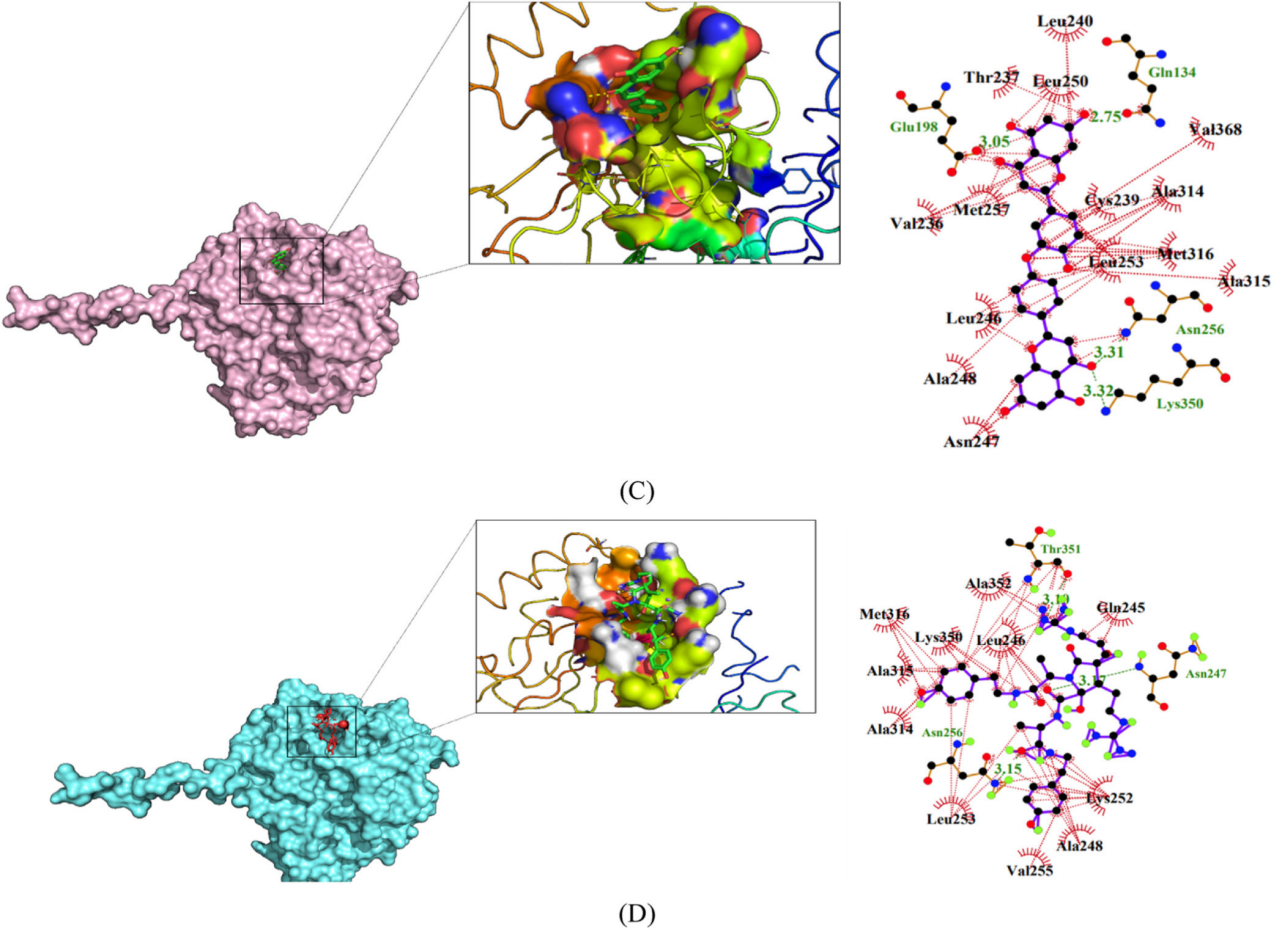

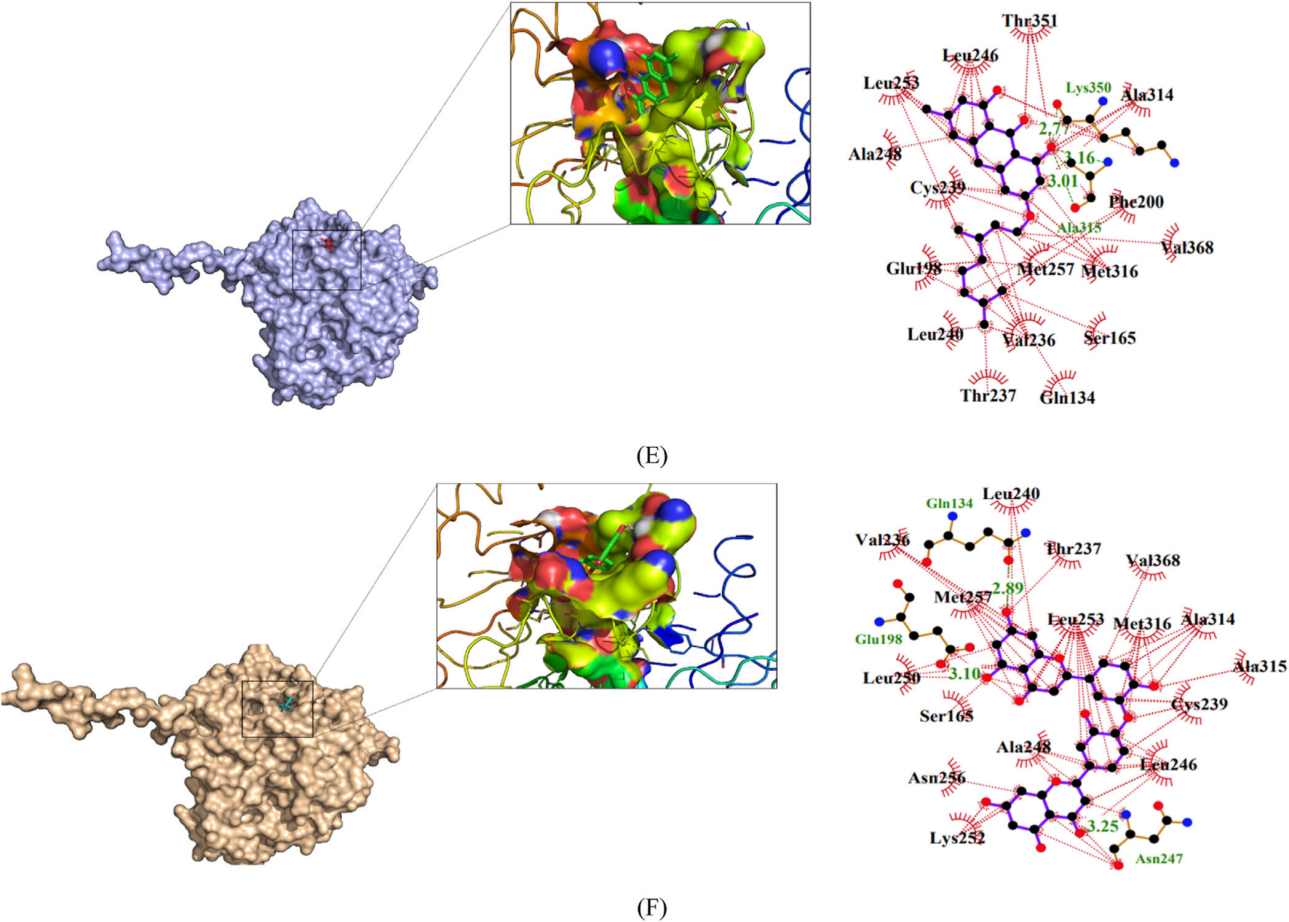
Binding mode evaluation of (A) Orthidine A, (B) S,5Z,8Z,11Z,13E,17Z)-15-hydroxy-1-(2,4,6-trihydroxyphenyl)-15-methylicosa-5,8,11,13,17-pentaen-1-one, (C) ZINC28462577, (D) anchinopeptolide A, (E) ZINC14760755, and (F) ZINC95486263 protein-ligand complexes. The figure shows the ligand represented as sticks docked inside the binding pockets with a surface representation. The Ligplot+ representations of the protein-ligand complex interactions are also shown. The hydrophobic contacts are shown as red spoke arcs whereas the hydrogen bonds are represented as short dotted green lines. (For interpretation of the references to colour in this figure legend, the reader is referred to the Web version of this article.)

Hydrogen bonding and hydrophobic interactions are critical in the stabilization of a ligand in the binding pocket of a receptor [79]. The protein-ligand interactions depend on the structure and functional groups of the ligand [79]. LigPlot+ [59] was used to generate the 2-D schematic representation of the protein-ligand interactions. A total of 25 out of 30 hits formed hydrogen bonds of varying lengths with residues of the active site. This suggests stabilization of the ligands in the binding pockets. Orthidine A formed the highest number of five hydrogen bonds with the residues (Fig. 7A, Table 3), denoting the high stability of Orthidine A in the binding pocket [80]. ZINC2842577 and S, 5Z,8Z,11Z,13E,17Z)-15-hydroxy-1-(2,4,6-trihydroxyphenyl)-15- methylicosa-5,8,11,13,17-pentaen-1-one formed four hydrogen bonds (Fig. 7B and C, Table 3). Moreover, three hydrogen bonding were observed anchinopeptolide A, ZINC14760755, and ZINC95486263 protein-ligand complexes (Fig. 7D, E and F, Table 3). The shortest hydrogen bond length of 2.64 Å was observed with robustaflavone. The shorter the hydrogen bond length, the stronger the bond [81]. Euphohelionon, anchinopeptolide A, ZINC95486082, and ZINC95486052 were the only compounds that did not interact with Phe167, Glu198 and Phe200 (Table 3). Euphohelionon formed one hydrogen bond of length 3.19 Å with Asn256 and hydrophobic intermolecular contacts with 10 residues namely Leu253, Ala248, Val255, Lys252, Ala352, Ala314, Lys350, Leu246, Gln245, and Thr351 (Table 3). ZINC95486082 formed eleven hydrophobic contacts and two hydrogen bonds (Table 3). ZINC95486052 interacted with the beta tubulin via hydrophobic interactions with residues namely Asn247, Thr312, Ala315, Val313, Leu253, Met257, Asn256, Lys350, Asn247, Lys252, Met316, Leu246, and Asn348. Table 3 shows the protein-ligand interactions of some 13 selected hits.

### 3.7. The anthelmintic activities of the hits

The anthelmintic related biological activity was predicted and reinforced using structural similarity evaluation with known anthelmintics. The main goal was to predict scaffolds that could be optimized for the development of anthelmintics inhibitors. PASS [60] was used to predict the pharmacological activities of the top 30 hits. The prediction was based on the structural-activity relationship between the compound of interest and a training set of over 26000 compounds with known biological activities [60]. The relevant biological activity considered in this study was the propensity of the hits to be anthelminthic. PASS was used in previous studies to predict the anthelmintic activity of novel compounds [82,83]. Experimental results from these studies mostly corroborated the PASS predictions, indicating that PASS is a plausible Bayesian-based technique for predicting the anthelmintic activity of a compound. For a given compound, PASS predicts Probable activity (P_a_) and Probable inactivity (P_i_), with both ranging between 0.000 and 1.000 for a predicted activity. A total of 19 compounds out of the 30 hits were predicted to be anthelmintic, with P_a_ > P_i_ (Table 4) which denote their likely anthelmintic potential [83]. The compound 6,10-dimethyl-9-methylene-2-(4-methyl-1,2-dioxabicyclo[2.2.2]oct-5-en-*l*-yl)undec-5-ene obtained from NANPDB had the highest P_a_ of 0.759 and P_i_ of 0.003 and with such P_a_ > P_i_, there is a high propensity for it to be a potent anthelmintic warranting further pharmacological evaluation [82,83]. Furthermore, a Drugbank similarity search [84] of 6,10-dimethyl-9-methylene-2-(4-methyl-1,2-dioxabicyclo[2.2.2]oct-5-en-*l*-yl) undec-5-ene was done to support the PASS predictions. The hit had a DrugBank similarity score of 0.511 to terpenin-4-ol, *an isomer of terpineol.* Terpine-4-ol has been reported to be an anthelmintic with potent activity against the eggs and larvae of helminth *Haemonchus contortus* [85,86]*. The DrugBank* [84] *performs structure similar searches by using locally developed SMILES string comparison method to identify related structures. The similarity scoring ranges from 0–1, with 1 being the exact compound.*

ZINC15120680 had the second highest P_a_ of 0.722 and P_i_ of 0.003. It also had a DrugBank similarity score of 0.599, 0.572 and 0.571 to Kaempherol, Quercetin and Genistein, respectively. Kaempherol, quercetin and genistein are naturally occurring products with multiple pharmacological actions, including anthelmintic activity [87]. Kaempherol and quercetin for instance were found to contribute to the anthelmintic property of 10 East African plants [88]. Moreover, these two natural products were reported to cause paralysis and death of known helminths *Taenia solium* (tape worm) and *Pheritima posthuma* (earthworm) [89]. Phytochemical analysis of the root tuber peels revealed rich genistein contents [87,90]. Genistein appears to have an eclectic mechanism of action against helminth parasites [91]. For instance, genistein was found to alter the carbohydrate metabolism of cestode parasites [87,92]. It also disrupts the spines and the tegumental surface of trematodes, leading to total paralysis or death. Moreover, genistein and its derivatives interfere with the microtriches, vesiculations and nuclear pyknosis of cestodes *E. multicularis* and *E. granulosus* [87,93].

ZINC95486052 and ZINC95486082 were structurally similar to naringenin with DrugBank similarity scores of 0.768 and 0.818, respectively. Naringenin was reported to have a synergistic effect on the synthesis and shedding of cuticle larval stages of helminth *H. contortus* [94]. A substructure probe via PubChem [52] revealed a common 2-phenylchromen-4-one substructure in ZINC15120680 and its similar DrugBank compounds (kaempherol, quercetin and genistein). ZINC95486052, ZINC95486082, ZINC28462577, ZINC95485922, ZINC14760755, robustaflavone, tetrahydrorobustaflavone and ZINC95485928 all had a 2-phenylchromen-4-one substructure. This substructure is native to the flavonoid family of natural products. Studies have also attributed the high anthelmintic property in flavonoids to the presence of reactive 2-phenylchromen-4-one structure [89].

ZINC95485928 was predicted to possess anthelmintic activity with P_a_ of 0.687 and P_i_ of 0.004. It had a DrugBank structural similarity score of 0.500 to Diosmetin. Diosmetin extracts from plant species *D. insularis* was found to be efficacious against gastrointestinal nematodes in goats [95]. The authors further suggested that flavones, a family of natural products could probably be responsible for anthelmintic activities [95]. Furthermore, the NANPDB hit Euphohelionon, which is a constituent of the *Euphorbia helioscopia* L. plant [96] was predicted to have an anthelminthic activity with P_a_ of 0.378 and P_i_ of 0.02. A study of the methanolic extracts of *E. helioscopia,* the parent plant of euphohelionon, revealed significant larvicidal and ovicidal effects against *H. contortus* worms, both *in vitro* and *in vivo* [97]. Table 4 summarizes the PASS anthelmintic predictions of the 19 hit compounds from AfroDB [28] and NANPDB [27].

### 3.8. Drug likeness and pharmacological profiling

Lipinski’s Rule of Five (RO5) [98] was used to evaluate the drug-likeness of the 19 predicted anthelmintic hits. The rule has been applied in analyzing African natural product libraries [28,99,100]. For a molecule to be qualified as “drug-like” and orally active, it must not violate more than one of the following criteria: a molecular weight ≤500 Da; logarithm of n-octanol/water partition ≤5, which determines the lipophilicity; hydrogen bond donors ≤5; and hydrogen bond acceptors ≤10. Table 5a provides the physicochemical properties and evaluation of the drug-likeness of the 19 predicted anthelmintic hits of which 12 did not violate any of the RO5 criteria. Furthermore, only three hits violated one of the RO5s. In total, 15 out of 19 hits were predicted to be druglike via SwissADME [61]. The remaining four hits with the least drug-likeness were tetrahydrorobustaflavone, robustaflavone, ZINC13480348 and euphohelionon.

Pharmacological profiling studies are important components of the drug development pipeline. The pharmacokinetic properties considered for this study included human intestinal absorption, Permeability Glycoprotein (P-gp) binding, blood-brain barrier and cytochrome P450 (CYPs450) inhibition [101]. A total of 10 hits were predicted to have high intestinal absorption. Permeability glycoprotein (P-gp) is an ATP-dependent efflux pump that moves substances from the intracellular space to the extracellular space [102]. P-gp has a broad substrate specificity [103] and the excretion of drugs back into the gut lumen by P-gp pharmacokinetically reduces the potency of some drugs which are P-gp substrates [104]. Moreover, studies have implicated P-gp as a contributing factor in helminth drug resistance [105]. Only three of the hits were predicted to be P-gp substrates. Another important pharmacokinetic parameter is the Cytochromes P450 (CYPs) inhibition. Cytochromes P450 (CYPs), which are major enzymes involved in drug metabolism belong to a protein super family containing heme [106]. Inhibition of CYPs may lead to possible drug-drug interactions [107]. The CYP1A2, CYP2C19, CYP2C9, CYP2D6 and CYP3A4 inhibition of the compounds were predicted via SwissADME (Table 5b). Toxicity predictions were also performed on the compounds due to their potential contribution to drug attrition [108]. The mutagenicity, tumorigenicity, irritancy and reproductive effect were predicted using Osiris Property Explorer (Table 5c). Furthermore, the nephrotoxicity and hepatotoxicity were predicted via ADVERPred, which utilized Structure−Activity Relationships (SARS) models built using the PASS [63]. The probability of activity (Pa) and probability of inactivity (Pi) for these parameters for the 19 compounds have been provided in Table 5c.

### 3.9. Molecular dynamics simulation of selected compounds

**Fig. 8.**
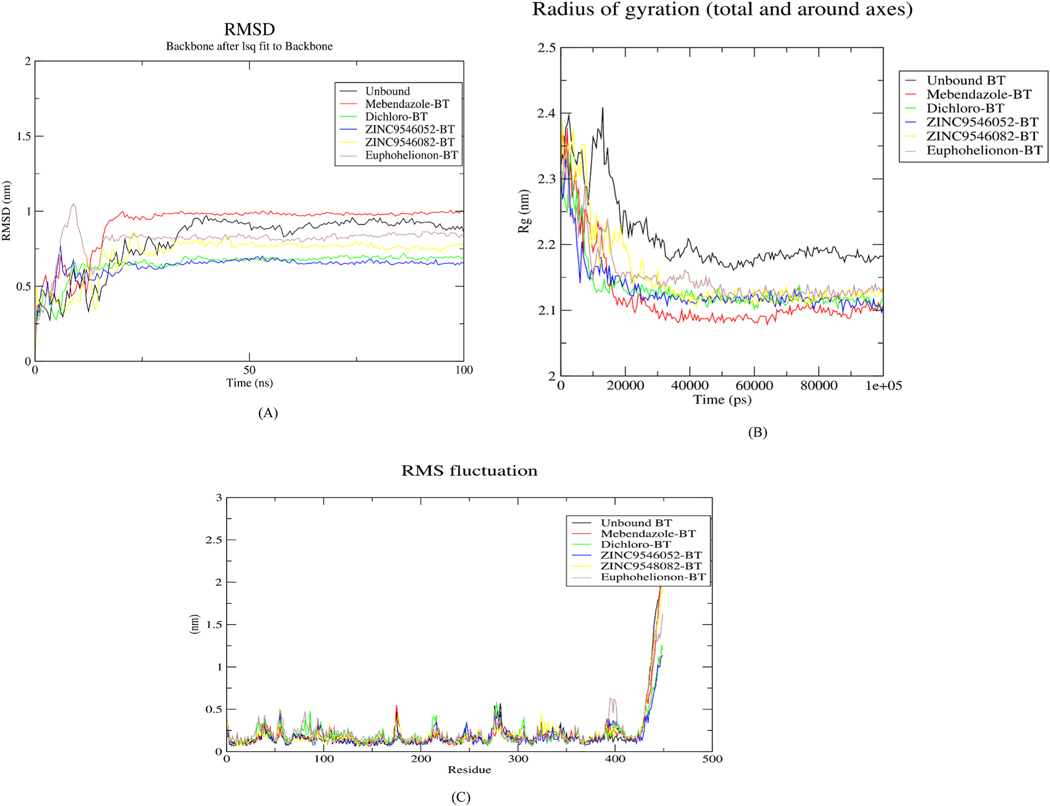
Graphical representations of Rg, RMSD, and RMSF of hookworm beta tubulin-ligand complexes over a 100 ns MD simulation. (A) Graph of backbone RMSD of the unbound protein and the respective complexes versus time in picoseconds. (B) The radius of gyration graph of the unbound protein and the other complexes versus time in picoseconds (C) Graph of RMSF of the unbound protein and the other complexes versus the number of residues. In all the three graphs (A, B and C), black, red, green, blue, yellow and purple plots represent the unbound protein, Mebendazole, PubChem ID 53327692 (Dichloro), ZINC9486052, ZINC9486082 and euphohelionon beta-tubulin complexes, respectively. (For interpretation of the references to colour in this figure legend, the reader is referred to the Web version of this article.)

Three African natural compounds namely ZINC95486082, ZINC95486052, and euphohelionon were selected for downstream MD simulation. These compounds had low binding energies, relatively good pharmacological profiles, predicted anthelmintic propensity and most importantly did not interact with the mutation-associated residues. Additionally, two known inhibitors comprising PubChem ID 53327692 and mebendazole were selected as controls. Both compounds had relatively better binding energies amongst the five inhibitors in this study. A 100 ns MD simulation was performed to expound on the structural stability and conformational changes when situated in diverse and dynamic physiological conditions [109]. The parameters studied included the root mean square deviation (RMSD), the radius of gyration (Rg), and the root mean square fluctuation (RMSF). The RMSD evaluates the deviation of the protein-ligand complex during the simulation from the initial protein backbone atomic coordinates and is used as a plausible measure of a protein’s stability [110]. An RMSD plot over simulation time revealed the backbones of the five complexes stabilized after 25 ns (Fig. 8A). The ZINC95486082-beta-tubulin complex rose from 0 nm to 0.5 nm during the start of the simulation. It then peaked around 23 ns, and later averaged around 0.75 nm over the remaining simulation time. Furthermore, the ZINC95486052-beta-tubulin complex maintained a steady RMSD of approximately 0.64 nm after the initial 20 ns of simulation. Additionally, the RMSD of Euphohelionon-beta-tubulin complex rose steadily from 0 nm and peaked around 1.0 nm, after which it began to decline. It then maintained an average RMSD of approximately 0.77 nm. The mebendazole-beta tubulin complex had the highest deviation of 1.0 nm from its protein backbone. The PubChem CID 53327692-beta tubulin complex also maintained an RMSD of approximately 0.64 nm.

The compactness of the complexes was evaluated using the Rg [111]. The Rg of all five complexes decreased over the first 20 ns. The trend then stabilized over the remaining simulation time (Fig. 8B). ZINC95486082–beta tubulin, ZINC95486052–beta-tubulin, Euphohelionon–beta-tubulin, Mebendazole-beta tubulin and PubChem ID 53327692–beta-tubulin complexes had average Rg of approximately 2.1 nm, 2.13 nm, 2.12 nm, 2.14 nm, and 2.13 nm, respectively. A stably folded and compact protein maintains a reasonable steady radius of gyration throughout the simulation [112]. As indicated in Fig. 7B, the trends appeared steady indicating the protein remained stably folded after binding.

Finally, the stability of the individual residues was assessed using their RMSF plots [109]. All the complexes possessed similar residue fluctuations within the same regions, with little deviation from the unbound protein core (Fig. 8C). Sizeable residue fluctuations were observed in the regions from residue index 420–449 which might be the region with the highest flexibility.

### 3.10. Evaluation of potential leads using the MM-PBSA calculations

The binding free energies of the complexes were estimated using MM-PBSA calculations. The calculations address some limitations of current scoring functions [113]. Contributing terms to the binding free energy include the electrostatic, polar, non-polar, and van der Waals energies. The binding free energies were computed in terms of average energies and standard deviations. Mebendazole had a relatively low average binding free energy of −150.810 kJ/mol (Table 6), thus affirming its binding energy against hookworm beta-tubulin. In addition, PubChem ID 53327692 had binding energy of −122.239 kJ/mol. The energy contributions per individual residues to the binding was also evaluated, whereby residues contributing >5.0 kJ/mol or < −5.0 kJ/mol were considered critical [114]. The amino acid residue, Glu198 contributed 34.3977 kJ/mol and 30.2668 kJ/mol to ligand binding of mebendazole and PubChem ID 53327692, respectively (Supplementary Figure 5).

**Fig. 9.**
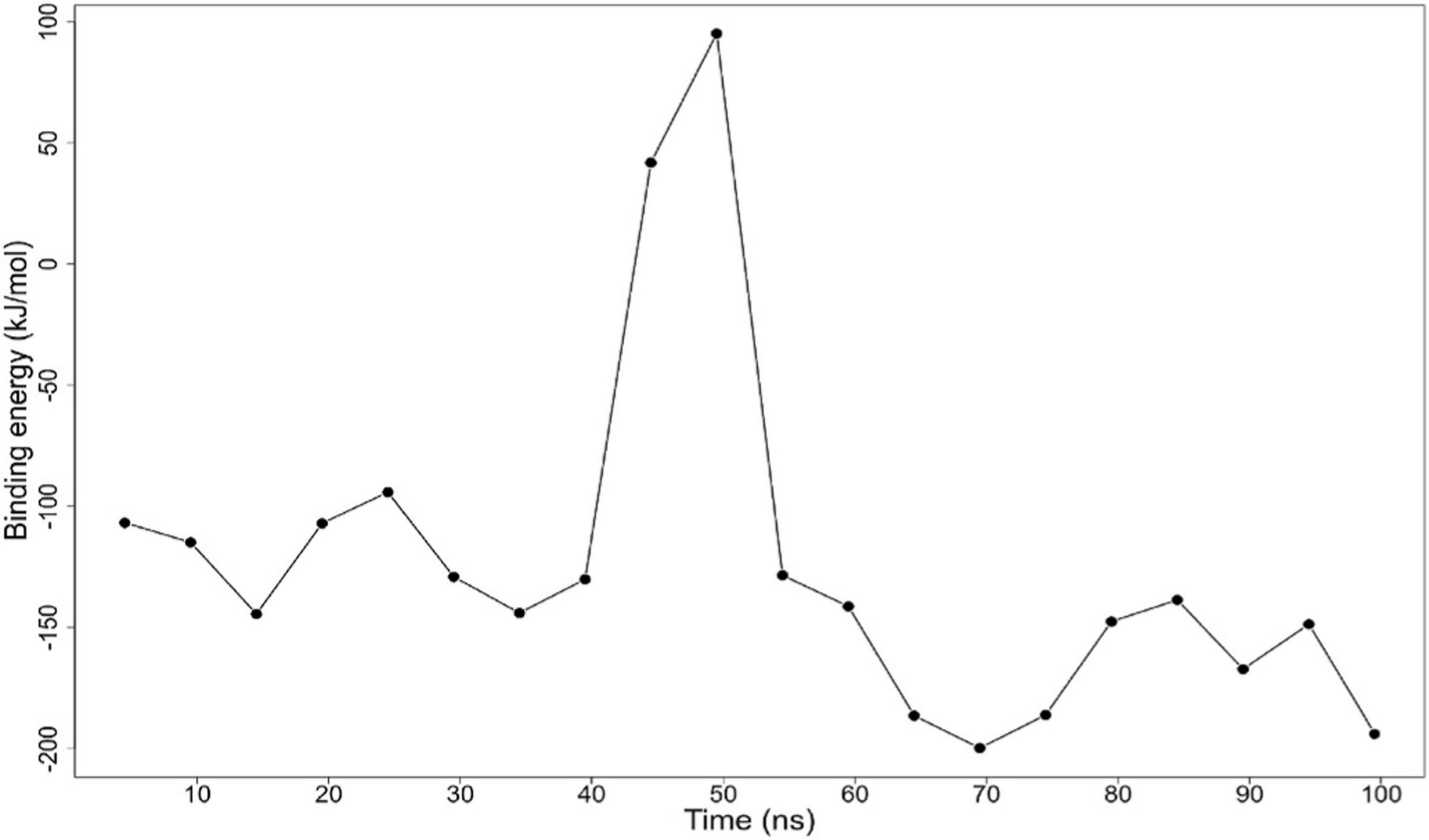
Binding energy plot of euphohelionon complex. The graph shows binding energy (kJ/mol) versus time over 100 ns simulation.

**Fig. 10.**
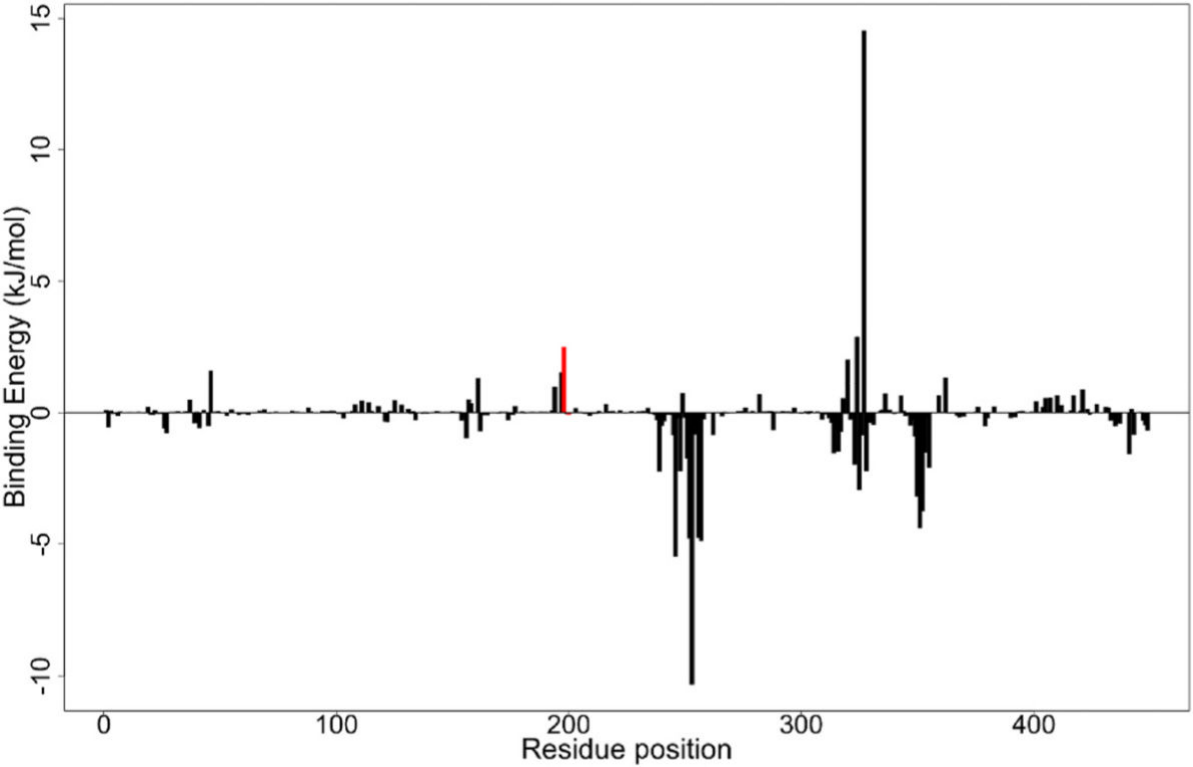
The per-residue decomposition of the binding energy plot of the euphohelionon-beta-tubulin complex. The per-residue binding energies of the likely mutated residues (Phe167, Glu198, and Phe200) are coloured red. The three residues contributed minimal energies to the complex. (For interpretation of the references to colour in this figure legend, the reader is referred to the Web version of this article.)

Euphohelionon had the lowest binding free energy of −123.620 kJ/mol amongst the three potential compounds (Table 6). Its binding energy plot over the simulation time showed initial binding energy of 106 kJ/mol. It then averaged within the range of −150 kJ/mol and −90 kJ/mol, further peaking at 50 ns. A final binding energy of −194 kJ/mol was obtained at 100 ns (Fig. 9). The van der Waal, electrostatic and polar energies contributed favourably to the free binding energy (Table 6). An energy decomposition plot revealed Leu246, Leu253 and Asp327 contributed energies beyond the ± 5.0 kJ/mol threshold (Fig. 10; Supplementary Table 2). The observed high energies corroborate the aforementioned assertion that Leu246 and Leu253 are critical for ligand binding and stabilization. Besides, the resistance mutation associated residues Phe167, Glu198 and Phe200 contributed energies of −0.0220 kJ/mol, 2.4911 kJ/mol and −0.0742 kJ/mol to ligand binding, respectively (Fig. 10; Supplementary Table 2). This fell short of the threshold required for critical residues, suggesting that perhaps these are not the foremost ligand-binding residues.

ZINC95486052 was predicted by AutoDock Vina to have binding energies of −8.5 kcal/mol. However, MM-PBSA calculations showed it had high binding free energy of 307.175 kJ/mol. This was predominantly due to high electrostatic and polar energies of 341.594 kJ/mol and 179.040 kJ/mol, respectively. Also, ZINC95486082 had high binding free energy of 336.564 kJ/mol. Molecules with high binding free energies may have limited lead likeness [114], hence a decreased propensity for further exploitation [115]. A binding energy decomposition per residue evaluation revealed Glu198 contributed favourably (>5.0 kJ/mol) to ligand binding of both compounds. Leu256 was observed to contribute favourable binding energies (< −5.0 kJ/mol) in both complexes.

### 3.11. Selection of parent molecule for de novo ligand design

The parent compound was selected based on its binding energy, protein-ligand interactions, predicted anthelmintic activity and free binding energies. After virtual screening an integrated library of 3385 compounds of African origin against the modelled beta-tubulin receptor, 30 hit compounds were selected based on their binding energies and how they are firmly docked in the binding pockets. All the selected hits had lower binding energy than the five known inhibitors. Compounds that form strong biomolecular interactions with resistance-associated mutation residues could lose interactions, leading to loss of affinity [21]. Four compounds namely anchinopeptolide A, euphohelionon, ZINC95486082 and ZINC 95486052 formed no molecular interactions with the resistance mutation associated residues (Table 3). However, anchinopeptolide A violated three rules of the RO5s (Table 5a). More so, it was not predicted as anthelmintic, hence warranted no further downstream exploration. ZINC95486082 and ZINC95486052 had binding of −8.5 kcal/mol and −8.3 kcal/mol, respectively. They were both predicted anthelmintics with Pa>0.3 and Pa>Pi (Table 4). Substructure searches revealed a common 2-phenylchromen-4-one substructure for ZINC95486082 and ZINC95486052. The 2-phenylchromen-4-one substructure was reported to be responsible for the high anthelmintic properties in flavonoids [89]. Both ZINC95486052 and ZINC95486082 were structurally similar to naringenin, a known anthelmintic with similarity scores of 0.768 and 0.818, respectively. However, evaluation of free binding energies of the two compounds via MM-PBSA calculations revealed high binding energies of 307.175 and 336.564 kJ/mol, respectively (Table 6), hence possibly decreasing their likelihood to be a lead compound [114].

**Fig. 11.**
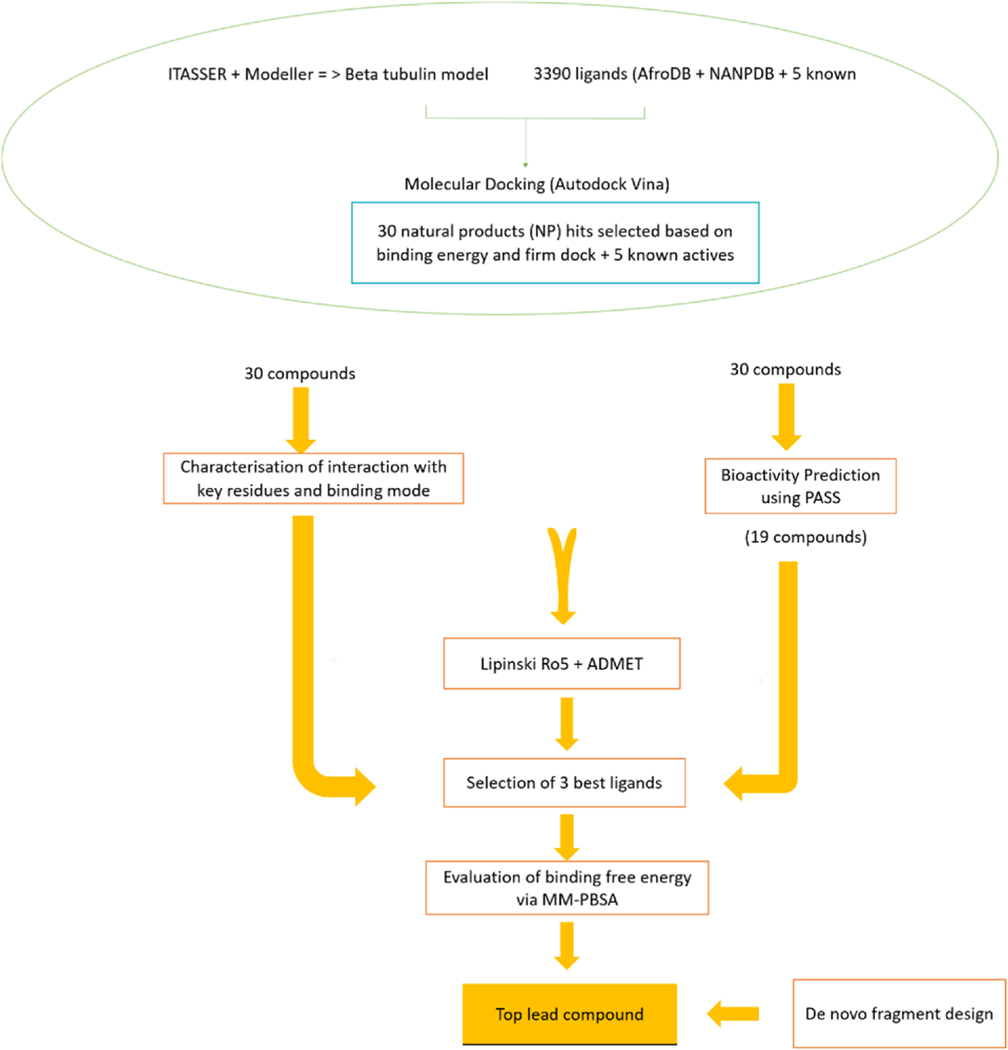
The schema shows the step-by-step approaches involved in the filtering and selection of compounds. Firstly, 3390 compounds comprising 3385 African natural products and 5 known beta-tubulin inhibitors were screened against the modelled beta-tubulin of the hookworm. Thirty compounds with low binding energies were selected as top hits for downstream analysis. The physicochemical and pharmacological properties, protein-ligand interactions and biological activity predictions of the 30 compounds were assessed *in silico.* Three natural product compounds were selected as leads based on their good pharmacological profiles, mechanism of binding, and predicted anthelmintic activity.

Euphohelionon had binding energy of −7.9 kcal/mol and formed one hydrogen bond with critical residue Asn256 and nine hydrophobic contacts. It was predicted as anthelmintic with a Pa of 0.378 and Pi of 0.02. When the Pa>0.3 and Pa>Pi for a particular biological activity, the compound warrants further experimental validation. Furthermore, euphohelionon is sourced from the *E. helioscopia* a local African plant, which has reported anthelmintic activities. Additionally, euphohelionon had a generally good pharmacological profile (Table 5). Further evaluation of the binding energies via MM-PBSA calculations revealed a good binding free energy of −123.620 kJ/mol (−29.546 kcal/mol). The energy contribution of the individual residues also revealed that three critical residues namely Leu246, Leu253 and Asp327, contributed energies beyond the ± 5.0 kJ/mol to the euphohelionon-beta-tubulin binding. Furthermore, per energy decomposition of the residues revealed it would likely not interact with the known resistance-associated mutation residues. Considering these factors, euphohelionon was selected as a parent compound for *de novo* design of potential inhibitor molecules of hookworm beta-tubulin. The step-by-step stages in the selection of a parent compound have been illustrated in Fig. 11.

### 3.12. De novo fragment-based design of novel potential anthelmintics

**Fig. 12.**
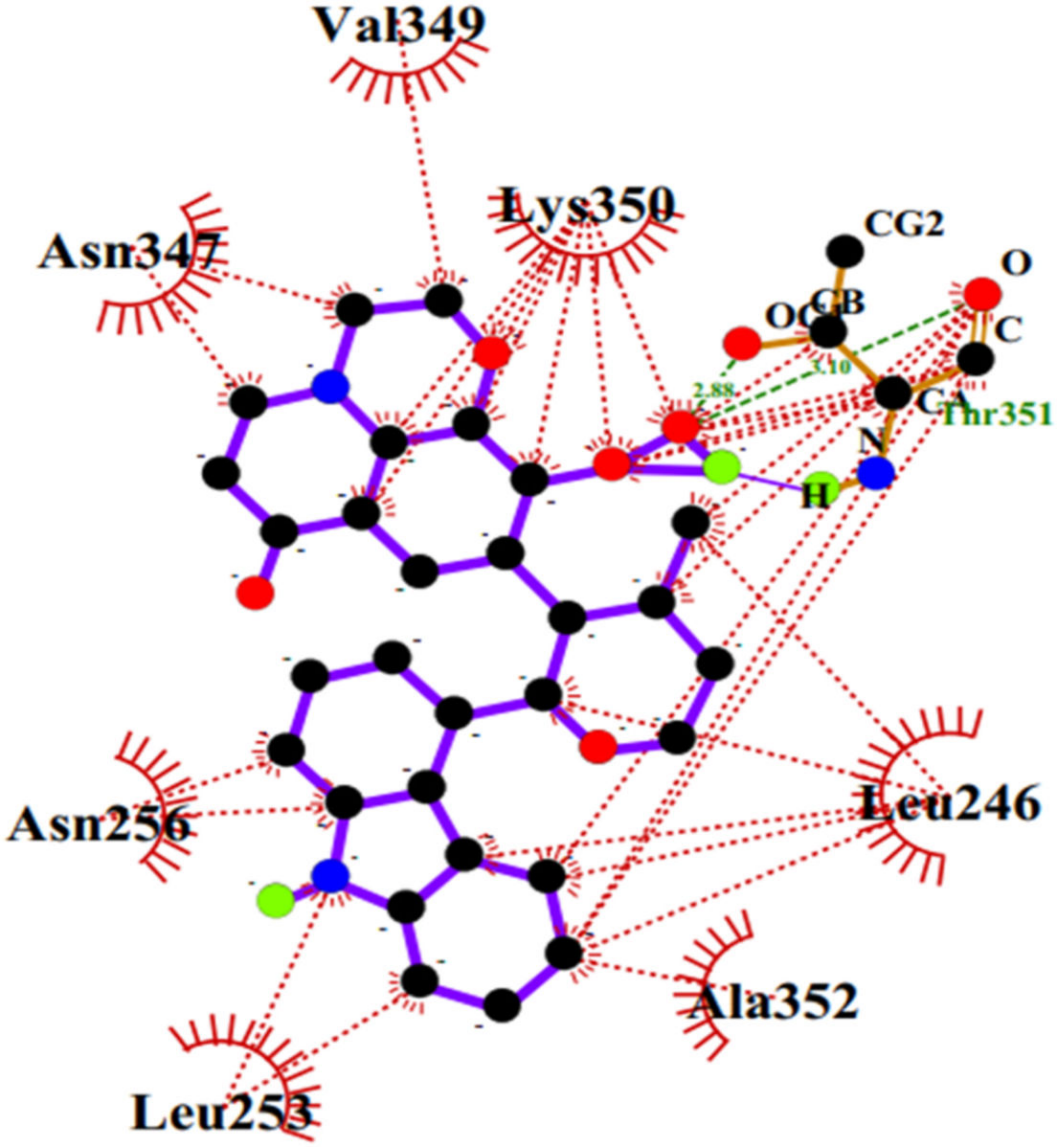
Ligplot+ representation of the protein-ligand interaction of the beta tubulin-A2 complex. The ligand forms six hydrophobic contacts (represented as red spook arcs) and two hydrogen bonding (represented as green dotted lines) with residues in the active site. (For interpretation of the references to colour in this figure legend, the reader is referred to the Web version of this article.)

The e-LEA3D server [67] was used to generate a total of 50 new molecules using euphohelionon as a parent compound. Duplicates and error generating molecules were eliminated. A total of 15 molecules with good physicochemical properties were used in molecular docking studies. The molecules with binding energies < −6.0 kcal/mol were selected for downstream analysis. Five novel molecules with identifications A1, A2, A3, A4 and A5 had good binding energies relative to the known inhibitors. Binding energies of −8.2 kcal/mol, −7.6 kcal/mol, −7.3 kcal/mol, −7.2 kcal/mol and −6.8 kcal/mol were obtained for A1, A2, A3, A4, and A5, respectively (Table 7). Only A1 had better binding energy than the parent compound, euphohelionon. Insights into the mechanism of binding revealed similar interactions akin to the euphohelionon-beta-tubulin complex. A1 formed a total of thirteen hydrophobic contacts with binding site residues including Leu246, Lys350 and Asn256. In addition, A2 formed two hydrogen bonds and six hydrophobic contacts (Fig. 12, Table 7). A3 formed nine hydrophobic contacts as well as three hydrogen bonds of varying lengths with Ala314. Furthermore, A4 formed two hydrogen bonds with Asn256 and Asn247. A5 formed a total of 11 hydrophobic contacts. Most importantly, none of the molecules interacted with the resistance mutation related residues (Table 7).

The novelty, physicochemical and pharmacokinetic profiles of the five compounds were assessed via SwissADME [61]. A search using the SMILES of the five compounds uncovered that all the molecules had no exact structure in the database, asserting their uniqueness. More so, all the molecules complied with R05s [98] except A1 which violated it. A1 violated two R05s comprising MW = 538.57 g/mol and logP = 4.35. Synthetic accessibility score (SAscore) is a useful parameter for estimating the ease of synthesizing a molecule. SAscore ranges from 1 (easy to synthesize) to 10 (difficult to synthesize). Molecules with SAscore *<*6.0 have lower propensity to synthesize [116,117]. Estimated SAscore values of 4.51, 4.94, 6.04, 3.1, and 3.22 were obtained for A1, A2, A3, A4 and A5, respectively. All the molecules were found to be within the acceptable threshold, except A3. Pharmacokinetic predictions also revealed all the molecules except A3 had high intestinal absorption (Supplementary Table 3). Undesirable pharmacological profiles of a compound can be improved through chemical modifications. Amongst the hits, A5 had no predicted toxicity (Supplementary Table 3). Additionally, anthelmintic activity predictions via PASS revealed A5 had a P_A_ of 0.448 and Pi of 0.028. When Pa>Pi and Pa>0.3, there is a high propensity for it to be a potent anthelmintic warranting further pharmacological evaluation [82,83]. Also, drug off-target activity as a result of drugs having different biological activities other than intended [118, 119] cannot be discounted. The two-dimensional structures of the five *de novo* molecules have been provided (Table 8).

## 4. Contribution to the field

Computational techniques have been proven to be a faster and cheaper means of exploring novel drugs. This study supplements current efforts by identifying three African natural compounds with good pharmacological profiles and most importantly predicted to have no interactions with the resistance-associated residues. The three-dimensional structure of the hookworm beta-tubulin was modelled with high confidence. Moreover, the *de novo* fragment-based design was employed to identify five unique compounds with the potential of circumventing the mutation associated residues. Additionally, a novel mechanism of binding was elucidated which could aid in the design of future potent hookworm beta-tubulin inhibitors.

## 5. Conclusion

This study identified three molecules with anthelminthic potential by screening a library composed of 3385 African natural products against a modelled 3D structure of the beta-tubulin gene product of *Necator americanus.* The AutoDock Vina molecular docking protocol was validated using the ROC with an acceptable AUC of 0.714. The three molecules, ZINC95486082, ZINC95486052 and euphohelionon, were predicted to have distinct mechanisms of binding relative to those of known drugs. These compounds did not interact with the benzimidazole resistance-related residues Phe167, Glu198 and Phe200 in *N. Americanus* beta-tubulin. A sub-structure analysis indicated that ZINC95486082 and ZINC95486052 contained 2-phenylchromen-4-one, a known anthelmintic moiety. Euphohelionon was selected as a parent compound for *de novo* ligand design because of its predicted anthelmintic properties, molecular interactions, and low binding energy consolidated with MM-PBSA calculations. Also, five novel molecules were generated using the fragment-based drug design and four can be synthesized easily. They also had favourable binding energies when compared to known inhibitors, and exhibited no contacts with any of the resistance mutation residues. These compounds can subsequently be synthesized and experimentally characterized for the development of much-needed novel anthelmintics.

## Supplementary Material

1

## Figures and Tables

**Table 1:** Comparison of DOPE, GA341 and molpdf scores of the five models predicted using MODELLER with the lowest discrete optimized protein energy (DOPE) score corresponded to Model 5.

Models	Molpdf score	DOPE score	GA341 score

Model 1	2642.68408	−52703.71875.	1.00
Model 2	2614.13916	−52903.56250.	1.00
Model 3	2478.50610	−52848.83594.	1.00
Model 4	2537.23853	−52964.20312.	1.00
Model 5	2550.17090	−53055.30859.	1.00

**Table 2:** Docking results for the top 30 hits and 5 beta-tubulin inhibitors. The table shows the IDs or common names, IUPAC names and their binding energies (kcal/mol) of the hits. The compounds are arranged in increasing order of binding energy. The lower the binding energy, the better the binding affinity. The IUPAC names were generated in MarvinSketch.

No.	ZINC ID/Common name/PubChem ID	IUPAC Name	Binding affinity (kcal/ mol)

	S,5Z,8Z,11Z,13E,17Z)-15-hydroxy-1-(2,4,6- trihydroxyphenyl)-15- methylicosa-5,8,11,13,17- pentaen-1-one	(5Z,8Z,11Z,13E,15S,17Z)-15-hydroxy-15-methyl-1-(2,4,6-trihydroxyphenyl)icosa-5,8,11,13,17- pentaen-1-one	−8.7
	ZINC14760755	3-{[(2E)-3,7-dimethylocta-2,6-dien-1-yl]oxy}−1,8-dihydroxy-6-methyl-9,10-dihydroanthracen-9- one	−8.6
	ZINC95485927	(3S,4aR,6aR,6bS,8aR,12aS,14aR,14bR)- 4,4,6a,6b,8a,11,11,14b-octamethyl- 1,2,3,4,4a,5,6,6a,6b,7,8,8a,9,10,11,12,12a,14,14a,14b- icosahydropicen-3-yl (2E)-3-(4-hydroxy- 3- methoxyphenyl)prop-2-enoate	−8.5
	ZINC95486082	(2S)-2-[2,2-dimethyl-8-(3-methylbut-2-en-1-yl)-3,4-dihydro-2H-1-benzopyran-6-yl]-7-hydroxy- 3,4-dihydro-2H-1-benzopyran-4-one	−8.5
	ZINC95486263	2-{4-[5-(5,7-dihydroxy-4-oxo-4H-chromen-2-yl)-2-hydroxyphenoxy]-3-hydroxyphenyl}−5,7- dihydroxy-4H-chromen-4-one	−8.5
	Campesterol	(1R,3aS,3bR,9aS,9bS,11aR)-1-[(2R,5R)-5,6-dimethylheptan-2-yl]-9a,11a-dimethyl-1H,2H,3H,3aH,3bH,4H,5H,5aH,6H,9H,9bH,10H,11H-cyclopenta[*a*]phenanthren-7-ol	−8.4
	ZINC14780716	(2E)-1-[2,4-dihydroxy-5-(3-methylbut-2-en-1-yl)phenyl]-3-[4-hydroxy-3-(3-methylbut-2-en-1-yl) phenyl]prop-2-en-1-one	−8.3
	ZINC95485922	2-[(2E)-3,7-dimethylocta-2,6-dien-1-yl]-1,3,5,8-tetrahydroxy-4-(3-methylbut-2-en-1-yl)-9H- xanthen-9-one	−8.3
	ZINC95486052	(2S)-2-[2,2-dimethyl-8-(3-methylbut-2-en-1-yl)-3,4-dihydro-2H-1-benzopyran-6-yl]-5,7- dihydroxy-3,4-dihydro-2H-1-benzopyran-4-on	−8.3
	ZINC95485928	7-[(2Z)-3,7-dimethylocta-2,6-dien-1-yl]-6,8,12-trihydroxy-2,2-dimethyl-2,5-dihydro-1,10- dioxatetraphen-5-one	−8.2
	Orthidine_A	N-[(2S,3S)-7-[(1E)-2-carbamimidamidoethenyl]-3-(3,4-dihydroxyphenyl)-2,3-dihydro-1,4- benzodioxin-2-yl]guanidin	−8.2
	Robustaflavone	6-[5-(5,7-dihydroxy-4-oxo-4H-chromen-2-yl)-2-hydroxyphenyl]-5,7-dihydroxy-2-(4- hydroxyphenyl)-4H-chromen-4-one	−8.2
	Tetrahydrorobustaflavone	6-[5-(5,7-dihydroxy-4-oxo-4H-chromen-2-yl)-2-hydroxyphenyl]-5,7-dihydroxy-2-(4- hydroxyphenyl)-4H-chromen-4-one	−8.2
	ZINC13480348	(2R)-7-{[(2E)-3,7-dimethylocta-2,6-dien-1-yl]oxy}- 5,10-dihydroxy-2-methyl-4-oxo-1,2,3,4- tetrahydroanthracen-2-yl acetate	−8.1
	Siphonellinol_C	(3R,5aR,6R,7S,9aR)-6-{2-[(5S,6S)-5-hydroxy-6-[(2E)-4-hydroxy-4-methylpent-2-en-1-yl]-2,6- dimethylcyclohex-1-en-1-yl]ethyl}−2,2,5a,7-tetramethyl-hexahydro-3H-1-benzoxepine-3,7-diol	−8.1
	ZINC28462577	2-{4-[5-(5,7-dihydroxy-4-oxo-4H-chromen-2-yl)-2-hydroxyphenoxy]phenyl}−5,7-dihydroxy-4H- chromen-4-one	−8.0
	ZINC95486072	(1R,4S,5R,8R,13R,14S,17S,18R)-4,5,9,9,13,20,20-heptamethyl-24 oxahexacyclo [15.5.2.0^1,18^.0^4,17^.0^5,14^.0^8,13^]tetracosane-10,22-dione	−8.0
	ZINC95486073	(1R,4S,5R,8R,13R,14S,17S,18R)-4,5,9,9,13,20,20-heptamethyl-24 oxahexacyclo [15.5.2.0^1,18^.0^4,17^.0^5,14^.0^8,13^]tetracosan-22-one	−8.0
	ZINC95486081	(2S)-7-hydroxy-2-[(2R,3S)-2-hydroxy-3-(3-methylbut-2-en-1-yl)-3,4-dihydro-2H-1-benzopyran-6- yl]-3,4-dihydro-2H-1-benzopyran-4-one	−7.9
	6,10-dimethyl-9-methylene-2-(4-methyl-1,2- dioxabicyclo [2.2.2] oct-5-en-*l*-yl) undec-5-ene	1-[(5E)-6,10-dimethyl-9-methylideneundec-5-en-2-yl]-4-methyl-2,3-dioxabicyclo[2.2.2]oct-5-ene	−7.9
	Spinescen	(5S)-3-[(13S)-13-hydroxy-13-[(2S,2^′^S,5S,5^′^R)-5’-[(1S)-1,5,6-trihydroxyundecyl]-[2,2^′^-bioxolan]- 5-yl]tridecyl]-5-methyl-5H-furan-2-one	−7.9
	Euphohelionon	(1R,3S,4S,5S,8R,10R,12R,13R,14S,15S)-8,15-bis(benzoyloxy)-3,7,11,11,14-pentamethyl-16- oxatetracyclo[11.2.1.0^1,5^.0^10,12^]hexadec-6-en-4-yl benzoate	−7.9
	Anchinopeptolide_A	(2S)-2-{[(2S,3R,4S)-3-(2-carbamimidamidoethyl)-4-(3-carbamimidamidopropyl)-2,4-dihydroxy-1-[(1S)-1-{[(1E)-2-(4-hydroxyphenyl)ethenyl]carbamoyl}ethyl]-5-oxopyrrolidin-2-yl] formamido}-N-[(1E)-2-(4-hydroxyphenyl)ethenyl]propanamide	−7.9
	ZINC33833639	(4aS,6aS,6bR,8aR,10S,12aR,12bS,14bS)-10-hydroxy-4a-(hydroxymethyl)-2,2,6a,6b,9,9,12a- heptamethyl-1,2,3,4,4a,5,6,6a,6b,7,8,8a,9,10,11,12,12a,12b,13,14b-icosahydropicen-5-one	−7.8
	ZINC95485992	(2E)-1-{2,4-dihydroxy-5-[(3S)-3-hydroxy-4-methylpent-4-en-1-yl]phenyl}−3-{4-hydroxy-3-[(3E)- 3-methylpent-3-en-1-yl]phenyl}prop-2-en-1-one	−7.8
	ZINC95486074	(1R,4S,5R,8R,10S,13R,14S,17S,18R,22S)-4,5,9,9,13,20,20-heptamethyl-24-oxahexacyclo [15.5.2.0^1,18^.0^4,17^.0^5,14^.0^8,13^]tetracosane-10,22-diol	−7.8
	ZINC95486075	(3S,4aR,6aR,6bS,8R,8aS,12aS,14aS,14bR)-8a-(hydroxymethyl)-4,4,6a,6b,11,11,14b- heptamethyl-1,2,3,4,4a,5,6,6a,6b,7,8,8a,9,10,11,12,12a,14,14a,14b-icosahydropicene-3,8-diol	−7.8
	ZINC13365959	3-[(1S)-1-(1H-indol-6-yl)-3-methylbut-2-en-1-yl]-6-(3-methylbut-2-en-1-yl)-1H-indole	−7.7
	ZINC13485435	(1R,10S)-6,13-bis(3-methylbut-2-en-1-yl)-8,17-dioxatetracyclo[8.7.0.0^2,7^.0^11,16^]heptadeca 2,4,6,11(16),12,14-hexaene-5,14-diol	−7.7
	ZINC15120680	2-[(2E)-3,7-dimethylocta-2,6-dien-1-yl]-1,3,5,8-tetrahydroxy-9H-xanthen-9-one	−7.7
	Mebendazole	methyl N-(6-benzoyl-1H-1,3-benzodiazol-2- yl)carbamate	−7.0
	PubChem CID 53327692	6,8-dichloro-2-{[(4nitrophenyl)amino]methyl} [1,2,4]triazolo[3,4b] [1,3]benzoxazole-3(2h)- thione	−6.4
	Albendazole	methyl N-[6-(propylsulfanyl)-1H-1,3-benzodiazol-2- yl]carbamate	−5.6
	PubChem CID 53327690	6,8-dichloro [1,2,4]triazolo[3,4-b] [1,3]benzoxazole-3(2h)-thione	−5.6
	PubChem CID 723308	5,7-Dichloro-1,3-Benzoxazole-2-Thiol	−4.8

**Table 3:** Protein-ligand interactions of some selected hits generated with Ligplot+. The table shows the binding energies, interacting residues in active binding site number of hydrogen bonds, with their bond length and the number of hydrophobic contacts.

ZINC ID/Compound Name	Binding energy (kcal/mol)	Interacting residues in the active site	Number of hydrogen bonds [amino acid residue (Å)]	Number of hydrophobic interactions	Total number of bonds

Orthidine A	−8.2	Asn256, Thr351, Asn348, Asn247, Ala248, Leu246, Lys350, Met257, Ala314, Ile345, Val313, Thr312	5 [Asn256: (3.02)] [Thr351: (3.13; 2.94)] [Asn348: (3.22)] [Asn247: (3.12)]	7	12
S,5Z,8Z,11Z,13E,17Z)-15-hydroxy-1- (2,4,6-trihydroxyphenyl)-15-methylicosa-5,8,11,13,17-pentaen-1-one	−8.7	Thr237, Gln134, Val236, Phe167, Phe200, Glu198, Ser165, Leu253, Asn256, Thr312, Asn348, Val313, Lys350, Met257, Leu246, Ala314, Cys239, Ala352, Met316, Ala248, Leu240, Val236	4 [Val236: (2.87)] [Glu134: (2.70)] [Glu198: (2.99)] [Asn256: (3.21)]	20	24
ZINC28462577	−8.0	Lys350, Asn256, Gln134, Glu198, Val236, Leu240, Leu250, Thr237, Met257, Leu246, Ala248, Asn247, Leu253, Met316, Ala314, Ala315, Val368, Cys239	4 [Glu198: (3.05)] [Gln134: (2.75)] [Asn256: (3.31)] [Lys350: (3.32)]	14	18
Anchinopeptolide A	−7.9	Val255, Lys252, Ala248, Leu253, Asn247, Ala314, Met316, Lys350, Leu246, Ala352, Gln245, Asn256, Thr351, Ala315	3 [Thr351: (3.10)] [Asn247: (3.17)] [Asn256: (3.15)]	10	13
ZINC14760755	−8.6	Ala248, Ala314, Met316, Met257, Leu246, Val368, Thr351, Ser165, Gln134, Phe200, Thr237, Leu240, Glu198, Cys239, Leu253, Lys350, Ala315	3 [Lys350: (2.77)] [Ala315: (3.01; 3.16)]	14	17
ZINC95486263	−8.5	Glu198, Gln134, Asn247, Ser165, Leu250, Met257, Leu240, Thr237, Leu253, Val236, Met316, Ala314, Val236, Val368, Cys239, Ala315, Leu246, Ala248, Lys252	3 [Glu198: (3.10)] [Gln134: (2.89)] [Asn247: (3.25)]	16	19
ZINC95485922	−8.3	Leu250, Val368, Phe200, Ser165, Glu198, Val236, Cys239, Asn247, Ala248, Leu246, Asn256, Lys350, Val313, Thr312, Ala314, Leu253, Met257, Asn348, Met257	3 [Asn247: (2.86)] [Lys350: (3.13)] [Asn256: (2.97)]	15	18
ZINC95486082	−8.5	Asn247, Ala315, Ala314, Cys239, Leu246, Met316, Leu253, Lys350, Asn256, Thr312, Val313	2 [Asn247: (3.32 Å; 3.07)]	9	11
robustaflavone	−8.2	Ala352, Leu246, Val368, Ala315, Ala314, Cys239, Met316, Val236, Met257, Glu198, Phe167, Ser165, Gln134, Leu250, Phe200, Leu253, Asn256, Lys350	2 [Asn256: (3.21)] [Gln134: (2.64)]	16	18
euphohelionon	−7.9	Leu253, Ala248, Val255, Lys252, Ala352, Asn256, Ala314, Lys350, Leu246, Gln245, Thr351	1 [Asn256: (3.19)]	10	11
ZINC95486052	−8.3	Asn247, Thr312, Ala315, Val313, Leu253, Met257, Asn256, Lys350, Asn247, Lys252, Met316, Leu246, Asn348	0	13	13
6,10-dimethyl-9-methylene-2-(4-methyl- 1,2-dioxabicyclo [2.2.2] oct-5-en-*l*-yl) undec-5-ene	−7.9	Glu198, Leu250, Val236, Phe200, Ser165, Met257, Val368, Cys239, Met316, Ala315, Ala314, Lys350 Thr237, Leu253, Gln134, Leu240	0	16	16

**Table 4:** PASS anthelmintic predictions of the 19 natural product hits from AfroDB and NANPDB. The table shows the ZINC IDs or names of the compounds, their probability of activity (Pa) and the probability of anthelmintic inactivity (Pi).

No.	ZINC ID/Compound name	P_a_	P_i_	P_a_>P_i_

	6,10-dimethyl-9-methylene-2-(4-methyl-1,2- dioxabicyclo[2.2.2]oct-5-en-*l*-yl)undec-5-ene	0.759	0.003	Yes
	ZINC15120680	0.722	0.003	Yes
	ZINC95485928	0.687	0.004	Yes
	ZINC95485922	0.627	0.004	Yes
	ZINC14760755	0.58	0.004	Yes
	ZINC14780716	0.482	0.02	Yes
	ZINC13480348	0.474	0.008	Yes
	Robustaflavone	0.464	0.024	Yes
	Tetrahydrorobustaflavone	0.464	0.024	Yes
	ZINC95486263	0.461	0.024	Yes
	ZINC28462577	0.457	0.025	Yes
	S,5Z,8Z,11Z,13E,17Z)-15-hydroxy-1-(2,4,6- trihydroxyphenyl)-15-methylicosa-5,8,11,13,17-pentaen-1-one	0.39	0.018	Yes
	Euphohelionon	0.378	0.02	Yes
	ZINC95486052	0.376	0.02	Yes
	ZINC95485992	0.367	0.058	Yes
	ZINC95486082	0.316	0.032	Yes
	Spinescen	0.291	0.118	Yes
	ZINC13485435	0.24	0.172	Yes
	ZINC95486081	0.207	0.084	Yes

**Table 5a:** Physicochemical properties of the 19 predicted anthelmintic compounds comprising MW = Molecular weight, HBD=Number of hydrogen bond donors, HBA=Number of hydrogen bond acceptors, LOGP = Coefficient of partition, and the drug-likeness based on the number of Lipinski violations.

No.	Compound ID	MW (g/mol)	HBA	HBD	LOGP	VIO-LATIONS

	6,10-dimethyl-9-methylene-2-(4-methyl-1,2-dioxabicyclo[2.2.2]oct-5-en-*l*-yl)undec-5-ene	318.49	2	0	4.76	1
	ZINC15120680	396.43	6	4	1.98	0
	ZINC95485928	462.53	6	3	2.93	0
	ZINC95485922	464.55	6	4	2.93	0
	ZINC14760755	392.49	4	2	3.38	0
	ZINC14780716	392.49	4	3	3.7	0
	ZINC13480348	452.54	6	2	2.92	0
	Robustaflavone	538.46	10	6	0.25	2
	Tetrahydrorobustaflavone	538.46	10	6	0.25	2
	ZINC95486263	554.46	11	6	0.02	3
	ZINC28462577	538.46	10	5	0.52	1
	S,5Z,8Z,11Z,13E,17Z)-15-hydroxy-1-(2,4,6-trihydroxyphenyl)-15-methylicosa-5,8,11,13,17-pentaen-1-one	440.57	5	4	3.35	0
	Euphohelionon	648.78	7	0	6.05	2
	ZINC95486052	408.49	5	2	2.9	0
	ZINC95485992	436.54	5	4	3.28	0
	ZINC95486082	392.49	4	1	3.46	0
	Spinescen	638.92	8	4	3.02	1
	ZINC13485435	392.49	4	2	3.73	0
	ZINC95486081	380.43	5	2	2.62	0

**Table 5b:** Prediction of the pharmacokinetics of the 19 predicted anthelmintic compounds showing gastrointestinal absorption (GI), Cytochrome P450 (CYP450) enzymes inhibition and permeability glycoprotein (P-gp) substratess

ZINC ID/Compound Name	GI Absorption	P-gp substrate	CYP1A2	CYP2C19	CYP2C9	CYP2D6	CYP3A4

6,10-dimethyl-9-methylene-2-(4-methyl-1,2dioxabicyclo[2.2.2]oct-5-en-*l*-yl)undec-5-ene	High	No	Yes	Yes	Yes	Yes	No
ZINC15120680	High	No	Yes	No	Yes	No	No
ZINC95485928	Low	No	No	Yes	No	No	No
ZINC95485922	Low	No	No	No	No	No	No
ZINC14760755	High	No	No	Yes	No	No	No
ZINC14780716	High	No	Yes	No	Yes	No	Yes
ZINC13480348	High	No	No	No	Yes	No	Yes
Robustaflavone	Low	No	No	No	No	No	No
tetrahydrorobustaflavone	Low	No	No	No	No	No	No
ZINC95486263	Low	No	No	No	Yes	No	No
ZINC28462577	Low	No	No	No	Yes	No	No
ZINC15120680	Low	No	Yes	No	Yes	No	No
S,5Z,8Z,11Z,13E,17Z)-15-hydroxy-1-(2,4,6-trihydroxyphenyl)-15-methylicosa- 5,8,11,13,17-pentaen-1-one	Low	No	No	No	Yes	No	Yes
Euphohelionon	High	No	No	No	No	Yes	No
ZINC95486052	High	No	No	Yes	Yes	No	Yes
ZINC95485992	High	Yes	No	No	Yes	No	Yes
ZINC95486082	Low	No	No	Yes	Yes	No	Yes
Spinescen	High	Yes	No	No	No	No	Yes
ZINC13485435	High	Yes	No	Yes	Yes	No	Yes

**Table 5c:** Toxicity predictions of the 19 predicted anthelmintic compounds using DataWarrior and ADVERPred. The mutagenicity, tumorigenicity, irritancy, reproductive effect, hepatotoxicity and nephrotoxicity were considered. ADVERPred predicts the probability of activity (Pa) and probability of inactivity (Pi).

ZINC ID/Compound Name	Mutagenic	Tumorigenic	Reproductive Effective	Irritant	Hepatotoxicity		Nephrotoxicity	
						
					Pa	Pi	Pa	Pi

6,10-dimethyl-9-methylene-2-(4-methyl-1,2-dioxabicyclo[2.2.2]oct-5-en-*l*- yl)undec-5-ene	None	High	None	None	None	None	None	None
ZINC15120680	None	None	None	None	None	None	None	None
ZINC95485928	High	None	None	None	None	None	None	None
ZINC95485922	None	None	None	None	None	None	None	None
ZINC14760755	None	None	None	None	None	None	None	None
ZINC14780716	None	None	None	None	None	None	None	None
ZINC13480348	High	None	None	High	None	None	None	None
Robustaflavone	None	None	None	None	0.522	0.184	None	None
tetrahydrorobustaflavone	None	None	None	None	0.522	0.184	None	None
ZINC95486263	None	None	None	None	0.711	0.096	None	None
ZINC28462577	None	None	High	None	0.722	0.092	None	None
ZINC15120680	None	None	None	None	None	None	None	None
S,5Z,8Z,11Z,13E,17Z)-15-hydroxy-1-(2,4,6-trihydroxyphenyl)-15-methylicosa-5,8,11,13,17-pentaen-1-one	None	High	None	High	None	None	None	None
Euphohelionon	None	None	None	None	None	None	0.244	0.232
ZINC95486052	None	None	None	High	None	None	None	None
ZINC95485992	None	None	None	None	None	None	None	None
ZINC95486082	None	None	None	None	None	None	None	None
Spinescen	None	None	High	None	0.384	0.271	0.369	0.103
ZINC13485435	None	None	None	None	None	None	None	None

**Table 6:** The MM-PBSA predicted scores for the ZINC95486052, ZINC95486082, euphohelionon, and PubChem ID 53327692 complexes. The table shows the average binding energies and their contributing terms in average ± standard deviations in kJ/mol.

Name	Electrostatic (kJ/mol)	Van Der Waals (kJ/mol)	Non-polar (kJ/mol)	Polar (kJ/mol)	Binding energy (kJ/mol)

ZINC95486052	341.594 ± 79.830	−195.548 ± 39.296	−17.911 ± 3.429	179.040 ± 88.856	307.175 ± 46.596
ZINC95486082	247.491 ± 55.392	−165.756 ± 71.527	−15.864 7.108	270.694 ± 93.924	336.564 ± 70.495
Euphohelionon	−68.456 ± 35.677	−186.699 ± 64.469	−15.841 ± 5.685	147.377 ± 47.152	−123.620 ± 70.683
Mebendazole	−81.725 ± 11.668	−217.842 ± 11.900	−17.814 ± 0.850	166.572 ± 15.425	−150.810 ± 14.230
PubChem ID 53327692	−76.394 ± 16.633	−246.863 ± 10.661	−20.181 ± 0.860	221.199 ± 29.753	−122.239 ± 21.460

**Table 7:** A summary of the protein-ligand interactions of the five novel compounds generated with Ligplot+. The table shows the binding energy, the synthetic accessibility score, the interacting residues in the active site, and the respective intermolecular bonds.

Compound	Binding Energy (kcal/mol)	Synthetic accessibility Score (SAscore)	Interacting Residues in the active site	Number of hydrogen bonds [amino acid residues (Ǻ)]	Number of hydrophobic bonds

A1	−8.2	4.51	Ala315, Met316, Leu246, Leu253, Thr351, Lys350, Cys239, Ala314, Asn256, Ala248, Asn247, Val25, Lys252	0	13
A2	−7.6	4.94	Val249, Asn347, Lys350, Asn256, Leu253, Ala352, Leu246, Thr351	2 [Thr351: 2.88; 3.10]	6
A3	−7.3	6.04	Asn247, Ala352, Ala248, Leu246, Lys252, Leu253, Asn256, Thr351, Ala314, Ala315, Lys350	3 [Ala315: 3.22; 2.92] [Lys350: 2.97]	
A4	−7.2	3.10	Lys252, Ala248, Leu253, Leu246, Lys350, Thr251, Asn247, Asn256	2 [Asn247: 2.14] [Asn256: 2.90]	
A5	−6.8	3.22	Val368, Ala315, Ala352, Ala314, Met316, Lys350, Thr351, Leu246, Leu253, Val236, Met257	0	11

**Table 8:** The names, generated IUPAC names via ChemAxon and two-dimensional chemical structures of the novel potential lead compounds generated using euphohelionon as a template via e-LEA3D [67].

Name	Generated IUPAC Name	Generated Chemical structure

A1	(2R)-11-[4-(9H-carbazol-4-yl)phenyl]-2-fluoro-6-hydroxy-7-phenyl-4-oxa-1-azatricyclo[7.3.1.0^5^, ^13^] trideca-5(13),6,8,11-tetraen-10-one	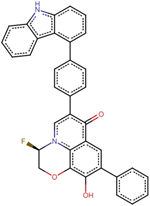
A2	7-[(2R,3R,4R)-2-(9H-carbazol-4-yl)-4-methyloxan-3-yl]-6-hydroperoxy-4-oxa-1-azatricyclo[7.3.1.0^5^, ^13^] trideca-5(13),6,8,11-tetraen-10-one	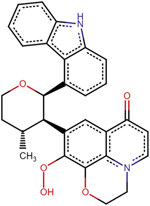
A3	(2S)-2-[(2R)-11-[(2S,3aR,6aR)-1-(9H-carbazol-4-yl)-octahydrocyclopenta[*b*]pyrrol-2-yl]-6-methyl-10-oxo-7-phenyl-4-oxa-1-azatricyclo[7.3.1.0^5^, ^13^]trideca-5(13),6,8,11-tetraen-2-yl]-2-amino-N-hydroxyacetamide	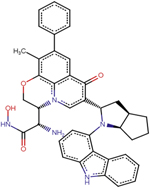
A4	7-(9H-carbazol-4-yl)-6-hydroxy-4-oxa-1-azatricyclo[7.3.1.0^5^, ^13^]trideca-5(13),6,8,11-tetraen-10-one	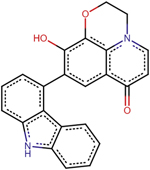
A5	(2S,3R)-2-(4-methylphenyl)-3-phenyloxane	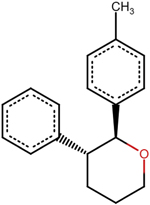

## List of Abbreviations

ADMET: Absorption, Distribution, Metabolism, Excretion and Toxicity
BBB: Blood-Brain Barrier
DOPE: Discrete optimized potential energy
FDA: Food and Drugs Authority
H-bond: Hydrogen bond
I-TASSER: Iterative Threading ASSEmbly Refinement
IVM: Ivermectin
MDA: Mass Drug Administration
MD: Molecular Dynamics
NP: Natural Product
NPs: Natural Products
PDB: Protein Data Bank
SDF: Structure Data File
SMILES: Simplified Molecular Input Line Entry System
RMSD: Root Mean Square Deviation
MMPBSA: Molecular Mechanics Poisson–Boltzmann Surface Area
