# Supplementary material for: Molecular modelling and de novo fragment-based design of potential inhibitors of beta-tubulin gene of Necator americanus from natural products
Source: Inform Med Unlocked. Author manuscript; Available in PMC 2021 Dec 14. (PMC8670734; doi:10.1016/j.imu.2021.100734)
Supplement: 1 [file NIHMS1753348-supplement-1.docx]

Supplementary Files

Supplementary Figure 1: Two-dimensional structures of the 5 known hookworm beta-tubulin used in this study.


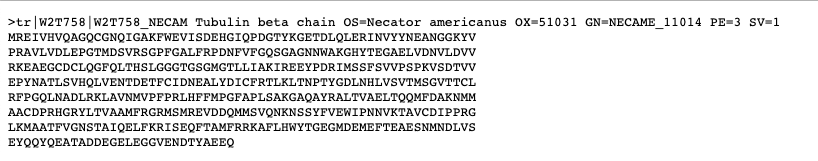


(A)


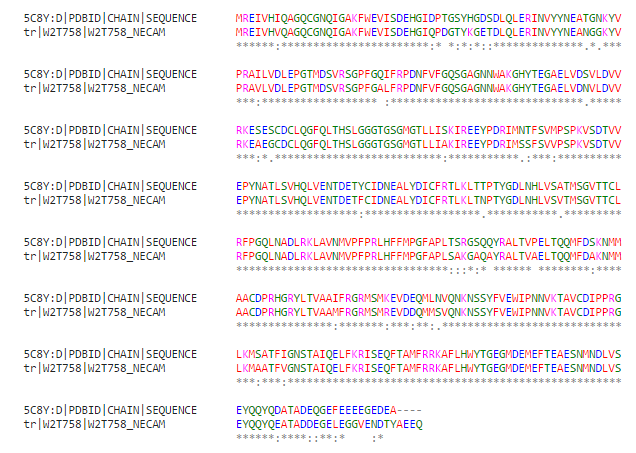


(B)

Supplementary Figure 2: (A) FASTA sequence of the *Necator americanus* beta tubulin gene. (B) A pairwise sequence alignment between the beta tubulin sequence of *N. americanus* and D chain of the crystal structure with PDB ID, 5c8y. The initials represent the amino acid residues. The PDB ID of the homologous template and the accession number of the beta tubulin of *N. americanus* are provided on the left. The highlighted residues supported with an asterisk (*) show the conserved residues between the sequences of the homologous template and *N. americanus* beta tubulin sequence.


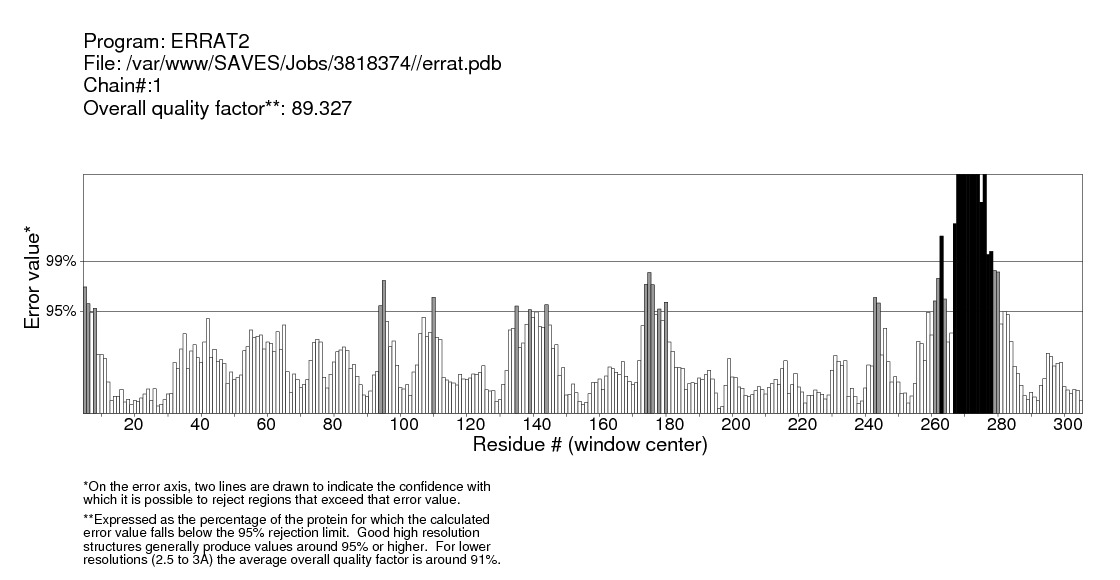


Supplementary Figure 3. Errat plot**.** Black bars identify the misfolded region located distantly from the active site, gray bars demonstrate the error region between 95% and 99%, and white bars indicate the region with a lower error rate for protein folding.


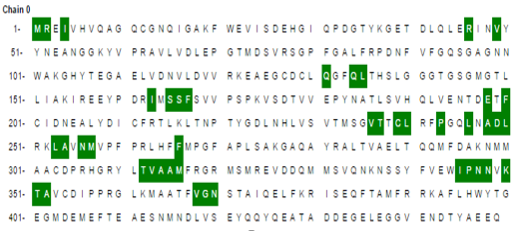


B. The amino acid residues of the colchicine-binding site in the modelled beta tubulin receptor of hookworm are shown in green.


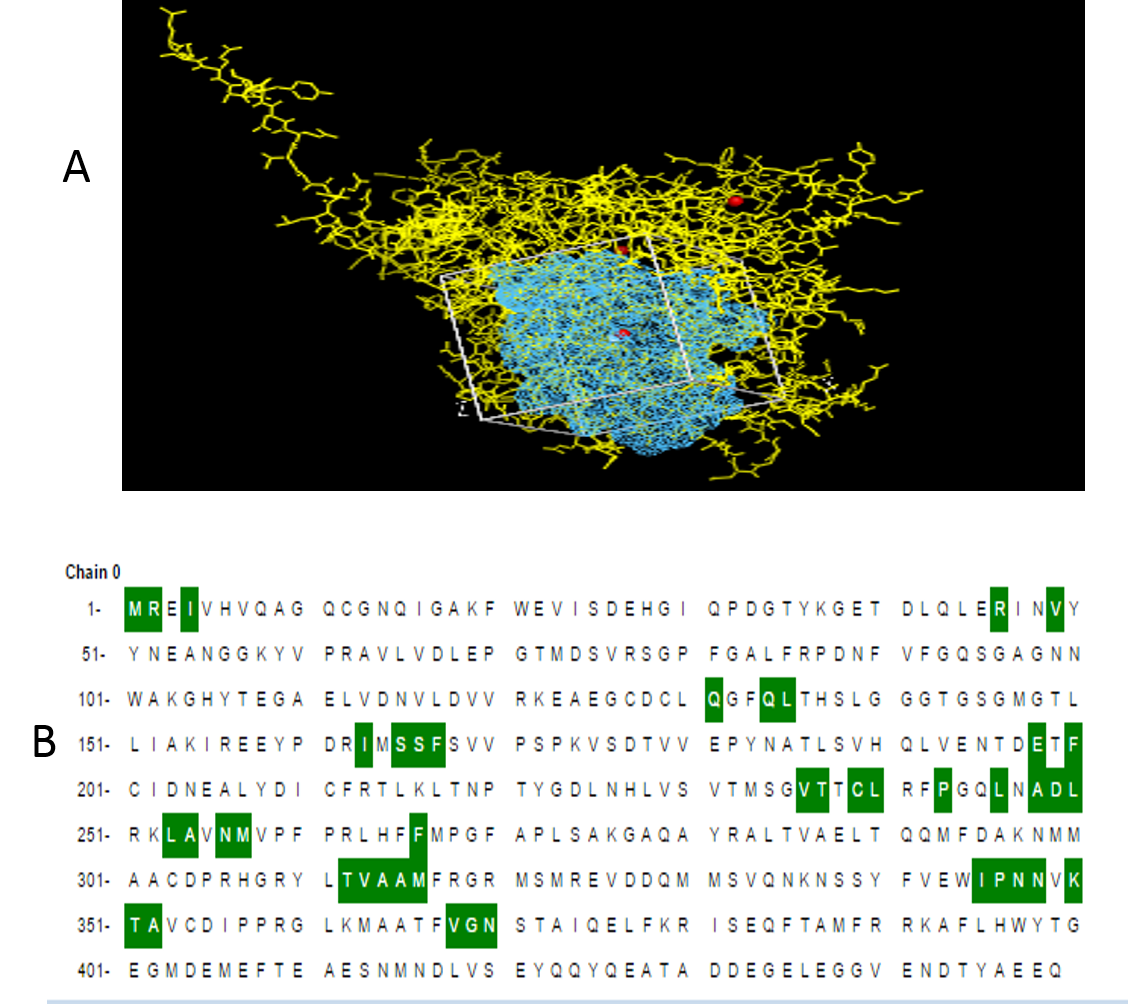


Supplementary Figure 4: Predicted colchicine binding site of beta tubulin from *N. americanus*. A. The binding pocket indicated in blue mesh surface and enclosed in a box is depicted in PYMOL. B. The amino acid residues of the colchicine binding site in the modelled beta tubulin receptor of hookworm are shown in green.


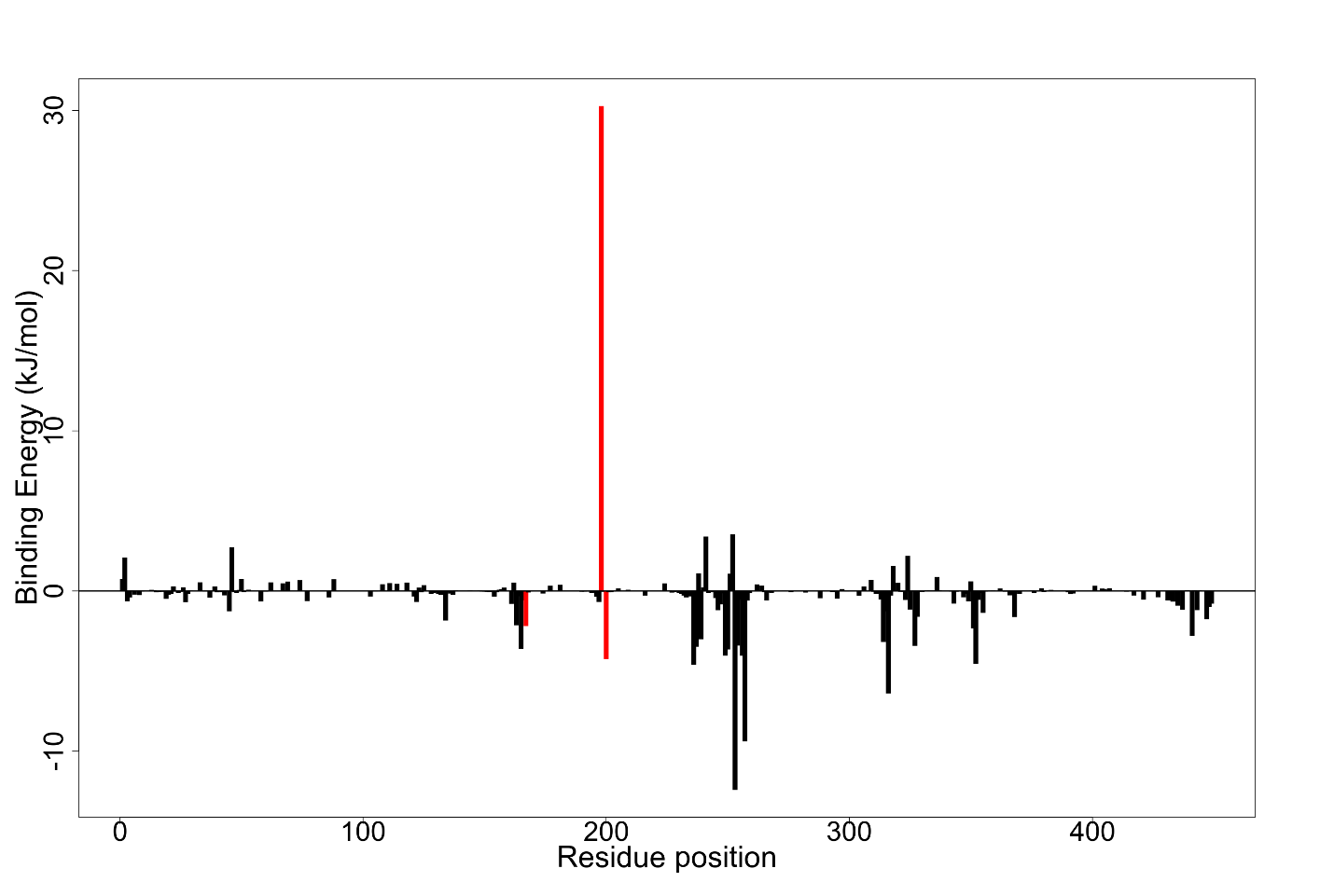


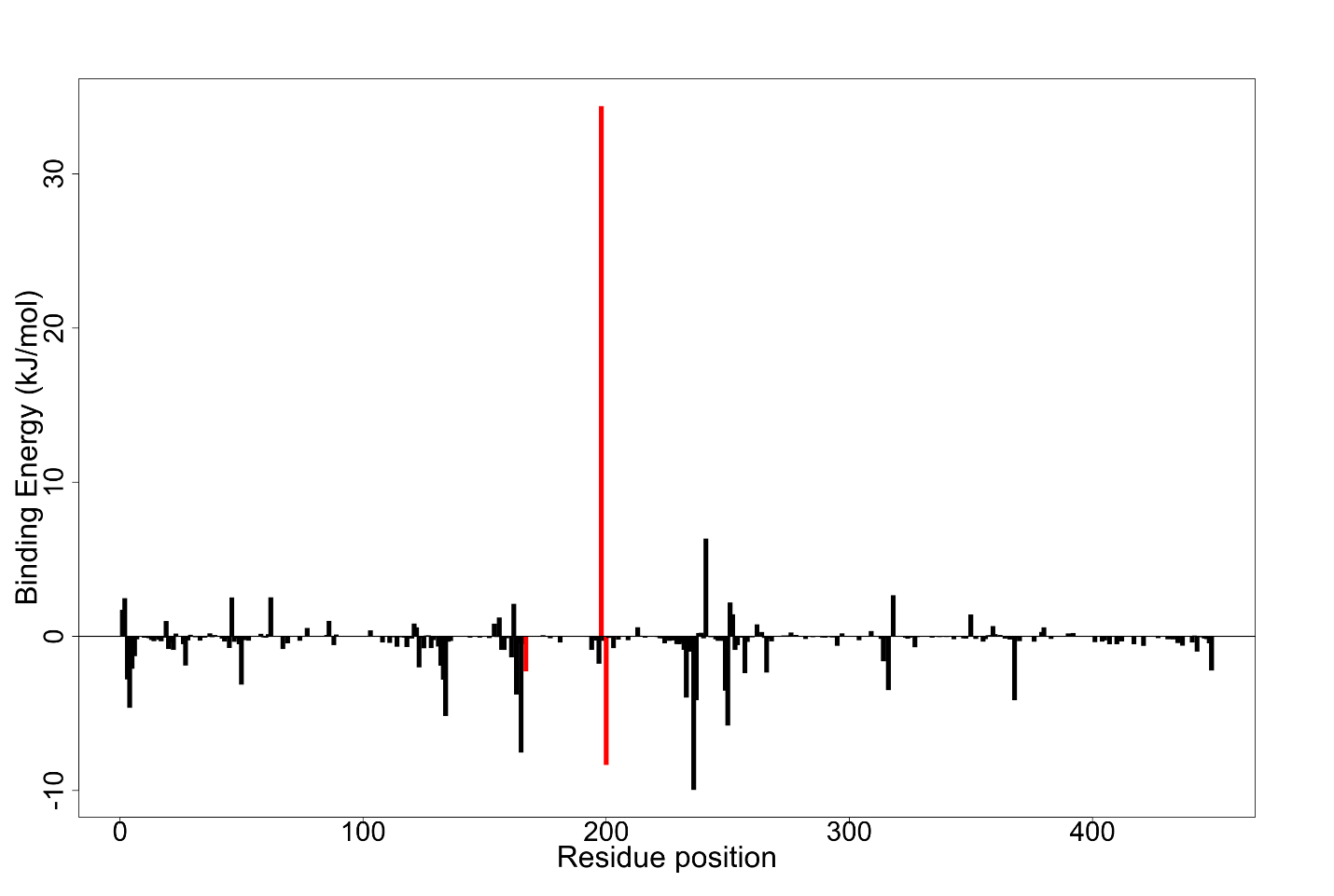


Supplementary Figure 5: The per-residue decomposition of the binding energy plot of (A) Mebendazole-beta tubulin (B) PubChem ID 53327692-beta tubulin complexes. The per-residue binding energies of the likely mutated residues (Phe167, Glu198, and Phe200) are colored red. The three residues contributed minimal energies to the complex.

Supplementary Table 1: Overview of computational tools used in the study

| Computational Tool | Use in study |
| --- | --- |
| I-TASSER^[31]^ | Template and binding site idenfication |
| MODELLER v.9.17 ^[32]^ | Generation of the 3-D structure of *N.americanus* beta tubulin using the D chain of the subunit of the multimeric structure of tubulin tyrosine ligase (T2R-TTL) [PDB ID: 5C8Y]. |
| WHAT IF server ^[34]^ | Fix steric clashes of model due to models having undesired bond lengths, bond angles, torsion angles and contacts |
| Swiss PDB viewer v 4.10 ^[35]^ | Correct local bond and angle geometry, and to relax the close contacts in the geometric chain of the modelled protein. |
| PyMOL v 1.74 ^[36]^ | Visualization of the protein model and protein-ligand complexes. |
| GROMACS v 5.1.4 ^[37]^ | Performing molecular dynamics simulation |
| PROCHECK v 3.5.4 ^[41]^ | Validation of modeled protein structure. A Ramachandran plot generated via PROCHECK highlighted the most favored, allowed, generously allowed and disallowed regions of the modelled protein structure. |
| CASTp  ^[44]^ | Predict putative binding pockets. |
| PyRx v 0.8 ^[55]^ | Virtually screen an integrated library of compounds against the modelled beta tubulin structure. |
| easyROC v 1.3.1 ^[58]^ | Generate the Area Under the Curve (AUC) of the Receiver operating characteristic (ROC) for validating the docking protocol. |
| Ligplot+ ^[59]^ | Study the 2D protein-ligand interaction which includes the hydrogen bonding and the hydrophobic contacts |
| PASS ^[60]^ | PASS predicts Probable biological activity (P_a_) and Probable biological inactivity (P_i_), with both ranging between **0.000 and 1.000** for a predicted activity. |
| SwissADME ^[61]^ | Evaluation of the physicochemical properties (molecular weight, number hydrogen bond acceptors and donors, logarithm of n-octanol/water partition) and pharmacokinetic properties (human intestinal absorption, Permeability Glycoprotein (P-gp) binding, blood-brain barrier and cytochrome P450 (CYPs450) inhibition) |
| DataWarrior v 4.5.2 ^[57]^ | Prediction of toxicity parameters namely mutagenicity, tumorigenicity, irritancy and reproductive effect. |
| ADVERPred ^[58]^ | Prediction of the probability of activity (Pa) and probability of inactivity (Pi) of the nephrotoxicity and hepatotoxicity of the compounds. |
| G_mmpbsa ^[61]^ | Compute the binding free energies of the complexes. |
| e-LEA3D ^[62]^ | Generation a total of 50 *de novo* molecules using euphohelionon as a parent compound |

Supplementary Table 2: A table showing the energy contributions of selected residues to ligand binding.

| Residue | PubChem CID 4030  (kJ/mol) | PubChemCID 53327692  (kJ/mol) | NANPDB2279  (kJ/mol) | ZINC95486052 (kJ/mol) | ZINC95486082 (kJ/mol) |
| --- | --- | --- | --- | --- | --- |
| Phe167 | -2.2841 | -2.1903 | -0.0220 | -0.1537 | -0.1481 |
| Glu198 | 34.3977 | 30.2668 | 2.491 | 19.5914 | 16.7503 |
| Phe200 | -8.3431 | -4.2438 | -0.0742 | -0.2494 | -0.2354 |
| Leu246 | -0.2966 | -1.1976 | -5.4780 | -2.5773 | -1.9587 |
| Leu256  Leu253 | -0.0787  -0.8914 | -4.0482  -12.4178 | -4.7666  -10.3391 | -3.0549  -0.3226 | -5.8900  -5.3785 |

Supplementary Table 3: Pharmacokinetics and toxicity predictions of the five selected *de novo* hits showing gastrointestinal absorption (GI), CYP3A4 (Cytochrome P450) enzyme inhibition, permeability glycoprotein (P-gp), mutagenicity, tumorigenicity, reproductive effect, and irritancy.

| Name | GI Absorption | P-gp substrate | CYP3A4  Inhibitor | Mutagenic | Tumorigenic | Reproductive Effective | Irritant |
| --- | --- | --- | --- | --- | --- | --- | --- |
| A1 | Low | No | No | High | High | None | None |
| A2 | High | Yes | No | Low | High | High | High |
| A3 | Low | Yes | Yes | High | High | None | None |
| A4 | High | Yes | No | High | High | None | None |
| A5 | High | Yes | No | None | None | None | None |
